# The promotion of well‐being among children exposed to intimate partner violence: A systematic review of interventions

**DOI:** 10.1002/cl2.1049

**Published:** 2019-09-30

**Authors:** Natasha E. Latzman, Cecilia Casanueva, Julia Brinton, Valerie L. Forman‐Hoffman

**Affiliations:** ^1^ RTI International Research Triangle Park North Carolina

## 1 PLAIN LANGUAGE SUMMARY

### 1.1 Limited evidence on the effectiveness of psychosocial interventions to promote well‐being among children exposed to intimate partner violence

Children's exposure to intimate partner violence (IPV) is a significant public health and social justice concern with potentially severe and long‐lasting effects. The extent to which psychosocial interventions promote well‐being among children exposed to IPV, and under what circumstances, such as the modality and setting, is unclear.

### 1.2 What is this review about?

Exposure to IPV childhood can have both short‐ and long‐term negative impacts to health and well‐being that persist across generations. There is therefore an increased interest in the development of intervention strategies to promote well‐being following exposure. Over the last two decades, theory‐driven psychosocial programs serving children exposed to violence have been developed and established in a range of venues (e.g., school‐based mental health clinics, outpatient psychotherapy settings). This review provides a synthesis of the state of this literature and implications for research and practice.

Specifically, we assess the effectiveness of psychosocial interventions in improving total problems, externalizing distress, internalizing distress, interpersonal/social problems, and cognitive functioning. We also consider variation in effects by intervention modality (e.g., individual, family‐based) and setting of the intervention (e.g., home, outpatient clinic).

What is the aim of this review?

This Campbell systematic review examines the effects of psychosocial interventions to promote well‐being among children exposed to intimate partner violence. The review summarizes evidence from eight methodologically rigorous randomized controlled trials.

#### 1.2.1 What studies are included in this review?

This review includes eight randomized controlled trials (RCTs), with a total of 924 participants.

The majority of studies were conducted in the United States, with one study each carried out in the Netherlands and India. The age range of target children varied, although all fell within the age range of 0–18 years.

Three studies recruited general populations of parents and/or children who had been exposed to IPV, without stated inclusion criteria around parent or child symptomatology or functioning. Four studies had more explicit inclusion requirements such as children with IPV‐related posttraumatic stress disorder (PTSD) symptoms and fathers with alcohol dependence. Studies varied widely regarding the nature of IPV experienced by parents and witnessed or heard by children.

### 1.3 What are the main findings of this review?

Studies examined following outcomes: total problems, externalizing distress, internalizing distress, interpersonal/social problems, and cognitive functioning. However, differences in the specific measures used, interventions employed, and comparison groups limit the ability to synthesize findings.

Evidence from two studies suggests there is preliminary evidence that in‐home intensive services (parent training and provision of emotional support to the parent) decreases child externalizing behavior among children who have been exposed to IPV and have clinical levels of behavior problems. However, support for this evidence was only found immediately posttreatment and at an 8‐month follow‐up, but not at a 4‐month follow‐up.

Intervention targeting the nonoffending parent (mother) had the largest effect, followed by those that targeted the family together and, finally, the single study that targeted parent and child, separately.

Interventions conducted in the home had a larger effect compared to those conducted in an outpatient setting. However, these findings should be interpreted with great caution due to the heterogeneity in study characteristics such as the nature of the comparators.

Overall, it is largely unclear the extent to which psychosocial interventions promote well‐being among children exposed to IPV, and under what circumstances.

### 1.4 What do the findings of this review mean?

The findings from this systematic review indicate that it is largely unclear the extent to which psychosocial interventions promote well‐being among children exposed to IPV, and under what circumstances. More rigorous evaluation of psychosocial interventions need to be conducted using common outcomes across studies in order to draw conclusions. We suggest that in addition to increased rigor in evaluation design (such as efforts to minimize selection bias), researchers assess the nature of child exposure and multiple subtypes of IPV; this will help elucidate whether interventions are more or less effective depending on the IPV exposure context.

### 1.5 How up‐to‐date is this review?

The review authors searched for studies up to April 2018.

### 1.6 Brief abstract

This systematic review examines the effects of psychosocial interventions to promote well‐being among children exposed to IPV. We searched 10 electronic databases and supplementary sources for studies published through April 2018. Eligible studies were those that employed an experimental or quasiexperimental design (with a comparison group); received a rating of low or moderate on a standardized risk of bias assessment; and examined child‐level outcomes (here defined as >75% of the sample between ages 0 and 17 years), regardless of the intervention target (e.g., child and/or parent). Eight methodologically rigorous RCTs, with a total of 924 participants, met criteria for inclusion in the review. Outcomes examined include overall child behavior, externalizing problems, and internalizing problems. Also examined are intervention modality (e.g., individual, family‐based) and setting of the intervention (e.g., home, outpatient clinic). Preliminary evidence from two studies suggests that in‐home intensive services decreases child externalizing behavior among children who have both been exposed to IPV and have clinical levels of behavior problems. However, the clinical and methodological variability of included studies precluded pooling of other trials. Thus, the findings from this systematic review indicate that it is largely unclear the extent to which psychosocial interventions promote well‐being among children exposed to IPV, and under what circumstances. The implications of these findings and recommendations for future research are discussed.

## 2 EXECUTIVE SUMMARY/ABSTRACT

### 2.1 Background

Children's exposure to IPV is a significant public health and social justice concern. The consequences of exposure can be severe and long‐lasting. Documentation of the immense magnitude and burden of children's exposure to IPV has been met with an increased interest in the development of intervention strategies to protect this vulnerable population and promote well‐being. Over the last two decades, theory‐driven psychosocial programs serving children exposed to violence have been developed and established in other venues (e.g., school‐based mental health clinics, outpatient psychotherapy settings). This review provides a synthesis of the state of this burgeoning literature and recommendations for research.

Specifically, we examined the impact of psychosocial interventions on well‐being among children exposed to IPV. Outcomes examined include overall child behavior, externalizing problems, internalizing problems. Also examined are intervention modality (e.g., individual, family‐based) and setting of the intervention (e.g., home, outpatient clinic).

### 2.2 Objectives

The objective of this review was to synthesize the evidence on the impact of psychosocial interventions on well‐being among children exposed to IPV.

### 2.3 Search methods

A combination of 10 databases and websites were searched. In addition to searching electronic resources, reference lists of relevant reviews (systematic and unsystematic) were scanned. Searches were executed by two reviewers and conducted between January and April 2018.

### 2.4 Selection criteria

Experimental and quasiexperimental designs (with a comparison group) were eligible for inclusion. Included studies must have received a rating of low or moderate on a standardized risk of bias assessment. Additionally, studies must have examined child‐level outcomes (here defined as >75% of the sample between ages 0 and 17 years), regardless of the intervention target (e.g., child and/or parent). All child‐level outcomes were of interest; that is, specific outcomes were not used as criteria for inclusion in the review.

### 2.5 Data collection and analysis

Our search identified 1,049 unique titles, all of which underwent abstract screening. Of these, 200 titles were retrieved for closer analysis of the full‐text based on the information included in the title and abstract. Subsequently, 169 full‐text reports were excluded that did not meet inclusion criteria leaving 31 articles for which we completed a risk of bias assessment. Finally, 16 articles (across 11 independent studies) studies were excluded due to coder assessed high risk of bias. This left 15 publications across eight independent studies in our final sample.

All included publications underwent systematic coding of study features. To examine the impact of interventions, all child‐level outcomes were converted into standardized effect sizes reflecting the direction and magnitude of intervention effects. If we found two or more similar studies for a comparison of interest, we conducted a meta‐analysis, under a random‐effect model, of the data from those studies. We report the impact of interventions using standardized differences of means, 95% confidence intervals (CIs), and respective forest plots. Subgroup analysis was conducted to examine the impact of characteristics (modality, setting) of interest. We assessed publication bias by constructing a funnel plot to display the precision versus effect sizes of each included study.

### 2.6 Results

We identified eight RCTs, with a total of 924 participants, reporting results on the impact of a range of interventions on well‐being among children exposed to IPV. Studies examined outcomes in the following domains: total problems, externalizing distress, internalizing distress, interpersonal/social problems, and cognitive functioning. However, the clinical and methodological heterogeneity of included studies largely precluded pooling of trials. Specifically, there was a high degree of heterogeneity with regard to differences in outcomes examined, interventions employed, and comparators (some studies employed a control group whereas others were comparative effectiveness studies that examined two active interventions).

Meta‐analysis was only able to be conducted for one outcome, externalizing behaviors. Meta‐analysis of two studies suggests there is preliminary evidence that in‐home intensive services (parent training and provision of emotional support to the parent) decreases child externalizing behavior among children who have been exposed to IPV and have clinical levels of behavior problems. However, support for this evidence was only found immediately posttreatment and at an 8‐month follow‐up, but not at a 4‐month follow‐up.

With regard to modality, pooled findings indicate that studies targeting the nonoffending parent (mother) had the highest pooled effect size, followed by those that targeted the family together and, finally, the single study that targeted parent and child, separately. With regard to setting, pooled findings indicated that studies conducted in the home had a larger pooled effect size as compared to those conducted in an outpatient setting. However, these findings should be interpreted with great caution due to the heterogeneity in study characteristics such as the nature of the comparators.

All findings taken together, it is largely unclear the extent to which psychosocial interventions promote well‐being among children exposed to IPV, and under what circumstances.

### 2.7 Authors’ conclusions

The findings from this systematic review indicate that it is largely unclear the extent to which psychosocial interventions promote well‐being among children exposed to IPV, and under what circumstances. Given the inconclusive findings from our review, below we outline three general conclusions and recommendations for future research to build the evidence base.

First, the evidence base remains underdeveloped and characterized by some breadth at the sake of depth. This breath became evident during our full‐text review and systematic application of inclusion and exclusion criteria. For example, 23 reports provided a program description or presented qualitative data only, and another 37 evaluated programs but failed to include a comparison group. This suggests there is great interest in developing, describing, and evaluating programs for children exposed to IPV. In addition, 20 reports were excluded because they did not present child‐level outcomes. Studies excluded for this reason typically examined outcomes at the level of the victimized parent. The addition of child‐level measures to parent‐focused evaluations would be a contribution to the evidence base and with relatively minimal resources. Finally, we found very few replication studies—our meta‐analyses of externalizing outcomes were in fact limited to a single replication study conducted by the same team as the original study. Paired with the fact that half of the studies in our review had fewer than 100 total participants, well‐powered replication studies conducted by independent research teams will undoubtedly help move the field forward.

Second, although 19 independent studies met our full‐text review inclusion criteria, 11 of these studies were ultimately excluded due to high risk of bias. Again, this highlights the breadth of programming, but unfortunately, lack of internal validity of a large proportion of existing work. We recommend—particularly for programs with strong theoretical grounding and uptake in the field (e.g., Kids’ Club, Pre Kids’ Club and Mom's Empowerment Program [MEP])—more rigorous evaluation by independent research teams.

Third, consistent with the larger literature, included studies generally failed to acknowledge the varied ways in which children come to know about their parent's IPV victimization (exposure) or consider the full range of the types of IPV to which children can be exposed. Future research should consider assessing the full range of ways in which children are exposed (direct involvement, direct eyewitness, indirect exposure) to multiple types of IPV (physical, sexual, and psychological aggression, and stalking).

## 3 BACKGROUND

### 3.1 The problem, condition, or issue

Children and adolescent's^1^ exposure to IPV, or domestic violence, is a pervasive public health problem. An estimated 8–15 million children in the United States (Hamby, Finkelhor, Turner, & Ormrod, 2011; McDonald, Jouriles, Ramisetty‐Mikler, Caetano, & Green, 2006) and 275 million children worldwide are exposed to IPV each year (Pinheiro, 2006). The consequences of exposure can be severe and long‐lasting. Research has linked IPV exposure in childhood to impaired neurological, physiological, and psychosocial functioning that contribute to a wide‐range of health consequences. Indeed, IPV exposure has been associated with reduced cognitive ability and educational achievement (Kitzmann, Gaylord, Holt, & Kenny, 2003), under‐immunization (Bair‐Merritt, Blackstone, & Feudtner, 2006), and both psychological (e.g., posttraumatic stress, depression, aggression; Evans, Davies, & DiLillo, 2008) and physical health problems (e.g., ischemic heart disease, obesity; Felitti et al., 1998). These negative developmental sequalae appear to be evident across nations and cultures; for example, the link between IPV exposure and future physical and/or sexual victimization has been found in studies conducted in the United States, China, South Africa, Colombia, India, Egypt, the Philippines, and Mexico; see Runyan, Wattam, Ikeda, Hassan, & Ramiro, 2002). Furthermore, recent work indicates that over an annual U.S. birth cohort of young adults, the health care, criminal justice and labor market costs associated with victimization amount to over $55 billion nationwide (Holmes, Richter, Votruba, Berg, & Bender, 2018).

Documentation of the immense magnitude and burden of children's exposure to IPV has been met with an increased interest in the development of intervention strategies to protect this vulnerable population and promote well‐being. Interventions for children exposed to IPV were initially developed in the late 1980s and 1990s and predominately focused on provision of general support; they were available only in battered women's shelters or from agencies providing services to victimized women (see Graham‐Bermann & Hughes, 2003 for a review of early programming). More recently, theory‐driven psychosocial programs serving children exposed to violence have been developed and established in other venues (e.g., school‐based mental health clinics, outpatient psychotherapy settings). A scan conducted almost 5 years ago identified 23 unique programs designed to improve outcomes for children exposed to IPV currently being implemented across the United States, with at least eight of these programs having been subject to one or more rigorous evaluations, including RCTs (Chamberlain, 2014).

### 3.2 The intervention

This review is focused on psychosocial interventions where the primary or secondary aim is the promotion of child well‐being following exposure to IPV. Psychosocial interventions are defined broadly to include a wide variety of services that emphasize psychological and/or social factors rather than biological factors. Interventions may be psychological in nature, such as psychotherapies of various orientations (e.g., cognitive‐behavioral or interpersonal therapy) *and/or* social in nature (e.g., peer support services) (England, Butler, & Gonzalez, 2015). Interventions may involve provision of psychosocial services to the exposed child, the exposed child plus a caregiver(s), or only a caregiver(s). Studies must include outcome data on at least one child‐level outcome to be included. Studies that report only caregiver‐level outcomes (e.g., parent depression) were excluded.

Interventions can occur in any setting, provided they include a psychological and/or social component; including but not limited to domestic violence shelters or service organizations, schools, outpatient clinics, criminal justice settings, or hospitals. The treatment modalities vary, including individual, family, or group‐based treatment. Below we provide example interventions in each of these categories.

#### 3.2.1 Individual intervention

One‐on‐one treatment permits attention to individualized traumatic cues, distorted thoughts, and behavioral interactions. For example, trauma‐focused cognitive behavioral therapy (TF‐CBT; Cohen, Mannarino, & Deblinger, 2006) is a clinic‐based intervention—rooted in learning and cognitive theories—addresses distorted beliefs and attributions related to the traumatic experiences and provides a supportive environment in which the child can talk about the traumatic experience or abuse. The treatment focuses on individual therapy sessions with children ages 3–18 years with parallel parent sessions that focus on the same elements as their child. Often, joint parent‐child sessions are also conducted. TF‐CBT is typically 12–16 sessions in length, although it has been modified into a shorter version for delivery in domestic violence shelters (Cohen, Mannarino, & Iyengar, 2011).

#### 3.2.2 Family‐based intervention

Family‐based interventions, such as child‐parent psychotherapy (CPP; Lieberman, 2004) involve sessions with a caregiver and a child age 0–5 years—with the dyad as the unit of treatment. CPP focuses on the focusing on the child–mother relationship as the therapeutic mechanism of change and usually delivered by therapists in 12–40‐hr‐long sessions. Whereas CPP was designed to address exposure to trauma broadly defined, Project Support (Jouriles et al., 2009) was developed specifically for mothers and their children, age 4–9 years, with a history of IPV exposure. This family‐based intervention is delivered by a therapist in the mother's home and focuses on increasing the mother's problem solving and behavior management skills. Project Support is typically involves 20 home visits over a 6‐month period.

#### 3.2.3 Group intervention

Group interventions, which typically are administered in schools, community settings, and domestic violence shelters, target general beliefs and attitudes about violence, reactions to violence, and social problem‐solving skills. For example, Kids’ Club and MEP (Graham‐Bermann, 2000; Graham‐Bermann, Lynch, Banyard, DeVoe, & Halabu, 2007) are two group‐based psychosocial programs delivered in a broad range of settings (e.g., community‐based agency, outpatient mental health clinic). Kids’ Club is designed for children age 6–12 years and creates a safe space for children to identify and express emotions and build social, emotional and coping skills. MEP is a 10‐session parenting group that provides support to mothers of children age 6–12 years by empowering them to discuss the impact of the violence on their child's development and to build parenting competence.

### 3.3 How the intervention might work

The potential pathway of effect between intervention and well‐being outcomes vary depending on the intervention—and specifically, the intervention's theoretical orientation. For example, MEP has an interpersonal relationship orientation. Based in part on Sullivan's (1953) interpersonal theory, the MEP emphasizes the whole person and explores strengths and abilities that can be used to compensate for relative weaknesses or psychosocial dysfunction. The groups were designed to provide a venue for exploring relationships, and specifically, by telling their stories and histories of IPV victimization, connecting events to emotional reactions, and enhancing self‐esteem, it is theorized that levels of traumatic stress will decrease, with subsequent improvements in parenting and child well‐being (Graham‐Bermann et al., 2007).

Interventions taking a cognitive‐behavioral orientation share the basic premise that psychological distress is maintained by maladaptive cognitions, or general beliefs about oneself and the world, contributing to specific and automatic thoughts in particular situations. The basic models holds that strategies to change these maladaptive cognitions leads to decreases in emotional distress and problematic behaviors and increases in well‐being. For example, TF‐CBT includes components teaching cognitive coping skills and cognitive restructuring (Cohen et al., 2006). Because at the onset of the current review the full range of orientations was not known (we searched the full range of possible psychosocial interventions), in descriptive tables we outline programs (and program orientations) tested in included studies.

### 3.4 Why it is important to do the review

Our primary goal was to systematically examine the available evidence for the impact of psychosocial interventions on well‐being following children's exposure to IPV. Here we use a broad definition of psychosocial interventions, as used by the Institute of Medicine of the National Academies, to include a wide variety of services (e.g., assessment, psychological counseling, group interventions, and education and support services that include a psychological and/or social component) that emphasize psychological and/or social factors rather than biological factors (England et al., 2015). Our secondary goal was to examine whether interventions with particular characteristics (e.g., modality, theoretical orientation) are more effective than others in promoting well‐being. Through this process, we aimed to identify gaps in the current scientific literature and highlight important areas for future research to build the evidence base.

Our review builds upon three primary literatures: (a) systematic reviews and meta‐analyses in related areas; (b) nonsystematic and critical reviews focused on interventions for children exposed to IPV; and (c) meta‐analyses focused on interventions for children exposed to IPV.

First, several systematic reviews and meta‐analyses have been conducted in related areas. Wethington et al. (2008) reviewed a range of interventions intended to reduce psychological harm from traumatic events—broadly defined—among children, adolescents, and young adults. This review did not separate out children's exposure to IPV from other types of trauma exposures (e.g., car accidents). Further, in 2013, two systematic reviews were commissioned by the Agency for Healthcare Research and Quality Effective Healthcare Program. In the first, Goldman et al. (2013) conducted a comparative effectiveness review of interventions addressing child maltreatment, and in the second, Forman‐Hoffman et al. (2013) conducted a comparative effectiveness review of interventions for children exposed to nonrelational traumatic events (e.g., accidents, natural disasters). Both of these reviews excluded evaluations of programs designed for children exposed to IPV, given the different nature of the trauma, resulting system responses, and intervention settings. For example, children exposed to IPV may not have access to resources traditionally offered to children engaged with the child protection system. Further, due to their own victimization, mothers experiencing IPV may be less able to provide support and stability to their own children (Lieberman & Van Horn, 2008); in fact, research indicates that mothers return to IPV perpetrators a mean of five times before permanently ending the abusive relationships (Sullivan & Bybee, 2004).

Second, several reviews have focused on interventions for children exposed to IPV. Rizo et al. (2011) conducted a critical analysis of interventions that either directly or indirectly target IPV‐exposed children; this review described programs and study designs but did not synthesize effects and therefore were unable to draw conclusions about the impact of interventions for children exposed to IPV. Futures without violence (Chamberlain, 2014) also conducted a scan of interventions designed for children exposed to IPV. The scan was focused on programs based in the U.S., and the authors did not systematically review and synthesize the results of empirical evaluations.

Finally, two meta‐analyses have synthesized the results of empirical evaluations of interventions designed to promote well‐being following exposure to IPV in childhood. However, these reviews differ from the current work in several important ways. First, Hackett et al. (2016) examined the overall impact of mental health‐focused interventions involving women and children in joint treatment. Results revealed effects in the medium to large range for child internalizing and externalizing symptomatology. However, this work included a broad range of study designs, including those without a comparison group. Furthermore, the authors did not assess risk of bias, thus potentially overestimating or underestimating the true intervention effect. Second, Howarth et al. (2016) conducted a mixed‐methods review of findings from studies that tested interventions to improve outcomes for children exposed to domestic violence and abuse published up through April 2013. The authors concluded that the evidence base, even from this comprehensive examination of evidence, lacked empirical data testing the clinical effectiveness, cost effectiveness, and acceptability of these interventions. Our review provides a more focused examination of impact with 5 more years of published literature.

Results of this systematic review have practice and policy implications. Given the immense magnitude and burden of children's exposure to IPV, there is a need for intervention strategies to protect this vulnerable population and promote well‐being. It is critical that practitioners and policymakers are provided with evidence‐based recommendations on the most efficacious interventions. Researchers may also use this work to help shape future studies, including, for example, selection of programs to subject to rigorous outcome evaluations, or to extend to a new population.

## 4 OBJECTIVES

### 4.1 The problem, condition, or issue

The objective of this review was to synthesize the evidence on the impact of psychosocial interventions on well‐being among children exposed to IPV. Our specific research questions were:

(1) Are psychosocial interventions targeting children who have been exposed IPV effective at promoting well‐being? Specific domains of well‐being include:
  a. Mental and behavioral health (e.g., posttraumatic stress symptoms, depressive symptoms; anxiety symptoms, adjustment problems, disruptive, aggressive, delinquent behavior, future victimization)
  b. Other development and school‐based functioning (e.g., cognitive development, academic achievement, social skills, executive functioning)
(2) Are interventions with particular characteristics more effective than others in promoting well‐being among children exposed to IPV? Specific intervention characteristics include:
  a. Modality (e.g., individual, family‐based)
  b. Theoretical orientation/approach (e.g., cognitive‐behavioral, interpersonal)
  c. Type of setting (e.g., domestic violence shelter, outpatient clinic).

## 5 METHODS

The title and protocol for the present review were registered in The Campbell Collaboration Library of Systematic Reviews in October 2016 and January 2018, respectively. Both are available at: https://campbellcollaboration.org/library/children‐partner‐violence.html

### 5.1 Deviations from protocol

This review deviated from the published protocol in several ways. These deviations are explained below.

(1) We intended to synthesize the literature on all child‐level well‐being outcomes; that is, outcomes measures were not used as criteria for inclusion in the review. However, as specified in the protocol, we expected studies to examine outcomes in the below categories:
  - Mental and behavioral health (including but not limited to posttraumatic stress symptoms, depressive symptoms, anxiety symptoms, disruptive, aggressive, delinquent behavior, future victimization)
  - Other development and school‐based functioning (including but not limited to cognitive functioning, academic achievement, and executive functioning)

Two outcomes of interest listed above, future victimization and school‐based functioning (e.g., academic achievement), were not examined in any of the studies included in the review. Further, we were only able to complete a meta‐analysis on externalizing outcomes. We were unable to identify enough comparable studies examining other mental and behavioral health outcomes (e.g., depressive symptoms) and other development (e.g., cognitive functioning) to complete meta‐analysis.

(2) We proposed a secondary aim examining the impact of theoretical orientation on intervention impact. This variable is included in the descriptive tables, but there was not enough heterogeneity in this variable to undertake a meaningful analysis.
(3) We planned to consider, analytically, study country/population's Human Development Index (HDI). Most included studies were conducted in the United States (with only two studies outside of the United States); there was not enough heterogeneity in this variable to undertake analysis.
(4) We proposed, and used, the tool developed by the Cochrane Collaboration (Higgins & Green, 2011) to assess risk of bias in RCTs. To assess risk of bias in observational studies, we added a second set of domains and subdomains from an item bank developed by RTI International (Viswanathan & Berkman, 2012) and a Cochrane Risk of Bias Assessment Tool for Nonrandomized Studies of Interventions (ACROBAT‐NRSI; Sterne, Higgins, & Reeves, 2014).
(5) Only studies rated to have an overall risk of bias of low or moderate were included in the final review. Studies rated as having a high risk of bias (or “critical bias” as described by Sterne et al., 2014) were excluded. Our decision to examine only studies assessed to be at low or moderate risk of bias was conservative, built from the concerns expressed by authors of prior reviews (e.g., Hackett et al., 2016; Howarth et al., 2016; Rizo *et al*., 2011) that conclusions around “what works” have been drawn from a literature dominated by studies that are not methodologically rigorous.

### 5.2 Criteria for considering studies for this review

#### 5.2.1 Types of studies

Both experimental and quasiexperimental designs with a comparison group were eligible for inclusion. Specific eligibility criteria are outlined below.

- RCTs in which individuals or parent‐child pairs are randomly assigned to intervention and comparison conditions;
- Quasiexperimental designs in which assignment to conditions is quasirandom, such as by birth date, day of the week, or another alternation method;
- Controlled quasiexperimental designs in which participants are not assigned to conditions randomly or quasirandomly, but in which participants self‐select into groups. Given the potential selection biases with controlled quasiexperimental designs, only the following types of quasiexperimental designs were eligible for inclusion: (a) regression discontinuity designs; (b) studies that use propensity score matching or other matching procedures to create intervention and comparison groups; or (c) studies in which participants in the intervention and control groups are not matched, enough statistical information must be reported that will allow estimation of preintervention effect sizes on at least one outcome.

Pre‐post designs without any comparison group and studies presenting only qualitative data were excluded.

#### 5.2.2 Types of participants

The specific population of interest is children and adolescents aged 0–17 years who have been exposed to IPV. Studies were included if >75% of the study population was in the age range of 0–17 years.

Of note, we used a broad definition of *exposure* to *IPV*, guided by Holden's (2003) taxonomy of exposure and the Centers for Disease Control and Prevention's (CDC) definition of IPV (see also, Latzman, Vivolo‐Kantor, Clinton‐Sherrod, Casanueva, & Carr, 2017):

- *Exposure*: Holden's (2003) comprehensive taxonomy of children's exposure to IPV outlines specific types of exposure, each falling into one of the four broad dimensions of prenatal exposure (e.g., mothers’ perception that the prenatal IPV had effects on their fetus), direct involvement (e.g., child intervenes during the incident), direct eyewitness (e.g., child sees the incident), and indirect exposure (e.g., child is exposed to the aftermath such as helping a parent with injuries). Here we focus on the latter three types of exposure (and not prenatal exposure) due to our focus on IPV exposure experienced between the ages of 0 and 17.
- *IPV*: The CDC outlines four subtypes of IPV that can be perpetrated by a current or former intimate partner: physical violence (e.g., slapping, use of a weapon), sexual violence (contact and noncontact behaviors such as rape, sexual harassment), stalking, and psychological aggression (expressive aggression and coercive control) (Breiding, Basile, Smith, Black, & Mahendra, 2015).

We excluded studies with children broadly identified as “at risk” for exposure (e.g., have experienced other types of victimization other than IPV exposure). Our review was international in scope and did not apply any restrictions related to nationality, language or cultural background.

#### 5.2.3 Types of outcome measures

##### 5.2.3.1 Primary outcomes

To prevent bias, outcomes measures were not used as criteria for inclusion in the review (Petticrew & Roberts, 2008). At the synthesis stage, only outcome measures addressing child well‐being were analysed. Any program implemented with IPV‐exposed children aged 0–17 years that intended to address the following well‐being outcomes (whether as a primary or secondary outcome) were included in the present review:

- Mental and behavioral health (including but not limited to posttraumatic stress symptoms, depressive symptoms, anxiety symptoms, disruptive, aggressive, delinquent behavior, future victimization)
- Other development and school‐based functioning (including but not limited to cognitive functioning, academic achievement, and executive functioning)

#### 5.2.4 Duration of follow‐up

All durations of follow‐up were included.

#### 5.2.5 Types of settings

All types of settings were included.

### 5.3 Search methods for identification of studies

#### 5.3.1 Electronic searches

Keywords derived from the study's inclusion criteria were combined using Boolean operators and used to identify potentially relevant studies (see below).

The first category lists key terms related to the population of interest: youth. The second category addresses exposure to IPV. The third category aims to identify “evidence focused” reports. In case the search capacity is limited only the term “evaluation” as be used as it is the most sensitive. Some databases allow choosing “RCT” as publication type, in which case the search was run twice with this option included. The intention of separating the terms in this manner was to include all the potentially relevant results, while simultaneously excluding the large bodies of literature on the impact of adult and youth exposure to IPV.

Our review intended to cover a broad‐range of outcomes related to well‐being, spanning mental and behavioral health to cognitive and school‐based functioning. Therefore, specifying key words related to well‐being was too limiting, and instead specific outcomes were retrieved and coded during full‐text review.

The following sets of keywords were combined with a Boolean AND:

*Age Group*: child* OR youth OR teen* OR minor OR kid OR juvenile OR adoles*

OR toddler OR baby

*Exposure*: (expos* OR witness* OR observe OR hear* OR see*) AND (fight* OR viol* OR aggress* OR assault OR abuse) AND (partner OR domestic OR parent* OR wife OR family OR mother OR boyfriend OR girlfriend)

*Outcome*: evaluation OR experiment* OR trial OR effective* OR efficacy OR RCT OR “random* control” OR quasi

The search strategy was applied to the following electronic databases, without restrictions on publication date: PubMed (Medline), PsycINFO, PsycARTICLES, Psychology and Behavioral Sciences Collection, Cumulative Index to Nursing and Allied Health Literature (CINAHL), Education Resource Information Center (ERIC), SocIndex with Full Text, Academic Search Premier, and ProQuest Dissertations and Theses. In addition, Google and Google Scholar were used to identify literature not formally published in sources such as books or journal articles (i.e., grey literature) using a combination of keywords and outcomes of interest. PubMed was also searched for publications ahead of print. All databases were searched from inception to the present date. Search results were merged using EndNote and duplicate records of the same publication/report were removed.

#### 5.3.2 Searching other resources

In addition to searching the electronic resources listed above, we reviewed reference lists from relevant review articles and included studies.

### 5.4 Data collection and analysis

#### 5.4.1 Data extraction and management

First, two independent reviewers (N. E. L. and J. B.) screened studies’ titles and abstracts. Second, potentially eligible studies—those marked as *potentially include* by either reviewer—were retrieved in full text and reviewed for eligibility against the inclusion criteria outlined above. Disagreements between reviewers were resolved via discussion and consensus, and if needed, consultation of a third study team member (C. C. and V. L. F.‐H.). Third, two reviewers (N. E. L. and V. L. F.‐H.) conducted a risk of bias analysis with disagreements resolved via discussion and consensus. In one instance, the team consulted an expert outside of the team. At this point, only those studies deemed at moderate or low risk of bias were maintained for inclusion in the review. Fourth, three reviewers (N. E. L., J. B., and C. C.) extracted the relevant data from each included article into the evidence tables. A second member of the team (N. E. L. and J. B.) reviewed all data abstractions for completeness and accuracy. Here again coding disagreements were resolved via discussion and consensus and on occasion, consultation of a third team member (V. L. F.‐H.).

Eligible studies were coded for a range of information relevant to study characteristics, intervention and comparison characteristics, and results (effect size data). Coding forms were managed in Microsoft Excel and transferred to Microsoft Word for presentation here (see Appendix A).

IBM SPSS Statistics 25 was used to conduct analyses focused on characterizing the features of studies with frequencies and other descriptive statistics.

#### 5.4.2 Assessment of risk of bias

To assess the risk of bias (internal validity) of studies, two independent reviewers used predefined, design‐specific criteria based on guidance in the Methods Guide (Viswanathan, Ansari, & Berkman, 2012). For RCTs, we used the risk of bias tool developed by the Cochrane Collaboration (Higgins & Green, 2011). This tool is a domain‐based evaluation in which critical assessments of high, low, or unclear risk of bias are made separately for the following types of bias and subdomains, (followed by current study benchmarks to be rated low risk of bias):

- *Selection*: Random sequence generation (random number generator used and is adequately described; use of medical record numbers or order of check‐in deemed inadequate); allocation concealment (clinician or researcher is unaware of or unable to predict the group to which any particular individual or family [i.e., the next one to enroll] will be assigned); baseline equivalence (groups similar at baseline; random sequence generation, if used, was not compromised prior to baseline data collection).
- *Detection*: Masked outcome assessors (outcome assessors unaware of or masked to conditions or purpose of the study).
- *Performance*: Participants blinded from allocated intervention.
- *Attrition*: Overall attrition (<20%); differential attrition (<15%), and methods for handling drop‐outs (owing to the weakness of each approach [see Higgins & Green, 2011, section 16.1.2], such methods may include, e.g., last observation carried forward, multiple imputation, statistical models to allow for missing data).
- *Reporting*: All prespecified outcomes reported.
- *Other*: Use of equal (applied to all participants), reliable and valid outcome measures; treatment fidelity (80% adherence based on measurement by independent evaluators).

Of note, performance bias is also typically assessed—although predominately in medical or pharmaceutical trials—by blinding personnel to the allocated conditions. In psychosocial interventions, it is often not desirable to blind the individual delivering the intervention. On the contrary, interventionists are often trained in the specific psychosocial therapy and expected to adhere to the program manual or intervention components (treatment fidelity).

Risk of bias in observational studies was assessed using questions from an item bank developed by RTI International (Viswanathan & Berkman, 2012) and a Cochrane Risk of Bias Assessment Tool for Non‐Randomized Studies of Interventions (ACROBAT‐NRSI; Sterne et al., 2014).

Here again we used a domain‐based evaluation in which critical assessments of high, low, or unclear risk of bias are made separately for the following types of bias and subdomains (followed by current study benchmarks to be rated low risk of bias):

- *Bias in Sample Definition and Selection*: Study design (prospective designs); recruitment source (participants in each group recruited from the same source location); recruitment time (participants in each group recruited over the same time period); inclusion and exclusion criteria equally applied in each group; and group differences considered in analyses.
- *Bias due to Confounding* (prognostic factors considered/controlled).
- *Bias in Interventions*: Interventions clearly defined across all study participants; no cross‐overs or contamination.
- *Bias in Measurement of Outcomes*: Masked outcome assessors (outcome assessors unaware of or masked to conditions or purpose of the study); use of equal (applied to all participants) reliable and valid outcome measures; time of follow‐up equal in all groups.
- *Bias due to Attrition or Missing Data*: Overall attrition (<20%); differential attrition (<15%); and methods for handling drop‐outs (owing to the weakness of each approach [see Higgins & Green, 2011, section 16.1.2], such methods may include, e.g., last observation carried forward, multiple imputation, statistical models to allow for missing data).
- *Bias in Reporting*: All prespecified outcomes reported.

Two trained members of the study team (N. E. L. and V. L. F.‐H.) independently rated the risk of bias for each study; conflicts were resolved by consensus, and in one instance, by consulting an expert outside of the study team. In general terms, a study with no identifiable flaws (with all subdomains receiving ratings of low risk of bias) was rated as having an overall low risk of bias. A study rated as having with moderate risk of bias was one deemed susceptible to some bias but probably not sufficient to invalidate its results. Although these studies had flaws in design or execution (e.g., >20% attrition), they provide information (e.g., through sensitivity analysis) to allow the reader the ability to evaluate and determine that those flaws did not likely cause major bias. Studies receiving an overall “high” risk of bias have at least one major flaw that likely caused significant bias, precluding the ability to draw causal inferences between the intervention and the outcome. When studies did not report sufficient detail to assess the validity of the design or study conduct, we judged the risk of bias to be unclear. Consistent with the Cochrane approach of prioritizing specific domains to determine overall risk of bias (see Cochrane Handbook, section 8.8.3.1), raters prioritized selection bias (RCTs) and bias in sample definition and selection (observational studies) when making overall rating determinations. Performance bias was considered a lower priority domain for determining overall risk of bias.

Only studies rated to have an overall risk of bias of low or moderate were included in the final review. Studies rated as having a high risk of bias (or “critical bias” as described by Sterne et al., 2014) were excluded. As noted by others (e.g., Higgins & Green, 2011; Rooney, Boyles, Wolfe, Bucher, & Thayer, 2014), more rigorous—and in this case, internally valid—studies are more likely to produce findings that are closer to the truth.

#### 5.4.3 Measures of treatment effect

As noted, we sought to examine a wide‐range of child‐level well‐being outcomes. To categorize results, we used an iterative process to create a hierarchical classification scheme that would represent key theoretical constructs and the scope of outcomes abstracted from the studies. The outcomes in the final coding scheme were classified into one of the following domains (and subdomains): (a) internalizing distress (depression, posttraumatic stress symptomatology); (b) externalizing distress; (c) total problems (e.g., Total Score on the Child Behavior Checklist) (d) interpersonal/social problems; and (e) cognitive functioning.

Included studies provided measures of treatment effects on both continuous and dichotomous scales. For all outcomes of interest, we calculated the standardized mean difference (SMD) across groups and the 95% CI of the standard error of the SMD effect sizes reported by each study author. When effect sizes were not reported by the study authors, but data needed to calculate an effect size were, we used the raw data for our calculations and then used methods in Lipsey and Wilson (2001) to calculate the SMD and 95%CI for each outcome. Examples of the types of data we used in our analyses for continuous outcomes include sample sizes in each group and (a) Cohen's *d* and 95% CIs; (b) pre‐ and postgroup means and standard deviations (SDs); (c) mean change and the SD of the mean change in each group; (d) group means and paired *t* statistics for each group; (e) group means and between‐group *t* statistic; and (f) between‐group *t* statistic only.

Examples of intervention effects that were reported on a continuous scale include internalizing and externalizing distress (e.g., behavioral checklist raw scores), total problems (e.g., behavioral checklists raw scores), and cognitive functioning (e.g., intelligence test total score). Examples of dichotomous outcomes included clinical levels of conduct disorder and of internalizing problems (e.g., diagnostic interview to assess presence or absence of meeting threshold for diagnosis of oppositional defiant disorder).

#### 5.4.4 Criteria for determination of independent findings

In the case of multiple outcomes within a study, we reported outcomes separately when possible (e.g., reporting depression and academic grades separately). When a single study reported results on multiple measures of the same outcome (e.g., two different measures of depression), we selected a single outcome based on a “pre‐defined hierarchy of outcomes” to reduce selection bias as suggested in the MEC2IR (2014) Standards. The elements of the hierarchy are that (a) we choose parent (or other‐reporter, such as a teacher) for externalizing symptomatology (e.g., disruptive behaviors) and (b) youth‐report for internalizing symptomatology (e.g., depression). If element (a) was insufficient to make a decision about which of multiple measures of an outcome to include, (b) we choose the measure with the greatest reliability and validity coefficients. If element (b) was insufficient (e.g., there was a tie or no reliability or validity information was available) we choose the measure randomly. If there were multiple groups, only those findings from the control groups and intervention groups which meet the eligibility criteria were included. In the case where the same results were reported in more than one report or manuscript, we designed the primary study as the one with the most complete data. For example, McFarlane et al. (2005b) reported 12‐month follow‐up data, whereas McFarlane, Groff, O'Brien, and Watson (2005a) reported the same outcome variables at 6, 12, 18, and 24‐month follow‐up periods. The latter report was designed as primary. Importantly, we calculated separate effect sizes separately for each follow‐up period.

#### 5.4.5 Unit of analysis issues

Studies based on the same sample were treated as a single study. As noted above, we coded the manuscript or report with the most complete information as the primary study; in instances where information was equally complete, the first dated study was designed as primary. Other studies from the sample were examined for additional information, including unique outcomes not reported in the primary study.

#### 5.4.6 Dealing with missing data

In the case of missing outcome data (i.e., not enough information was presented to compute a standardized effect size), we planned to contact study authors. However, all needed information to compute effect sizes was found in the published articles. We successfully contacted study authors in two other instances: to obtain information on follow‐up periods (Jouriles et al., 2001) and intervention settings (Lieberman, Van Horn, & Ippen, 2005).

#### 5.4.7 Assessment of heterogeneity

We required that studies had low clinical and methodological heterogeneity before determining pooling for meta‐analysis was appropriate. For example, the populations, interventions and comparators had to be similar, and the outcomes examined had to measure similar constructs at similar time intervals following the end of treatment. As part of the quantitative synthesis, we assessed the statistical heterogeneity in effects between studies by calculating the *I* ^2^ statistic, which corresponds to the proportion of variation in study estimates due to heterogeneity.

#### 5.4.8 Assessment of reporting biases

We assessed publication bias by constructing a funnel plot to display the precision versus effect sizes of each included study.

#### 5.4.9 Data synthesis

If we found two or more similar studies for a comparison of interest, we conducted a meta‐analysis of the data from those studies. We used random‐effects models to estimate pooled effects from studies we determined to be appropriately pooled.

#### 5.4.10 Subgroup analysis and investigation of heterogeneity

In addition, we conducted subgroup analyses among groups that study authors tested to determine if the efficacy of an intervention varied by a characteristic of interest. In each subgroup, we assessed the statistical heterogeneity in effects between studies by calculating the I2 statistic.

#### 5.4.11 Sensitivity analysis

As suggested by Higgins and Green (2011), we conducted a sensitivity analyses to examine whether the overall result of the analysis is robust in the use of imputed correlation coefficient.

## 6 RESULTS

### 6.1 Description of studies

#### 6.1.1 Results of the search

**Figure 1:**
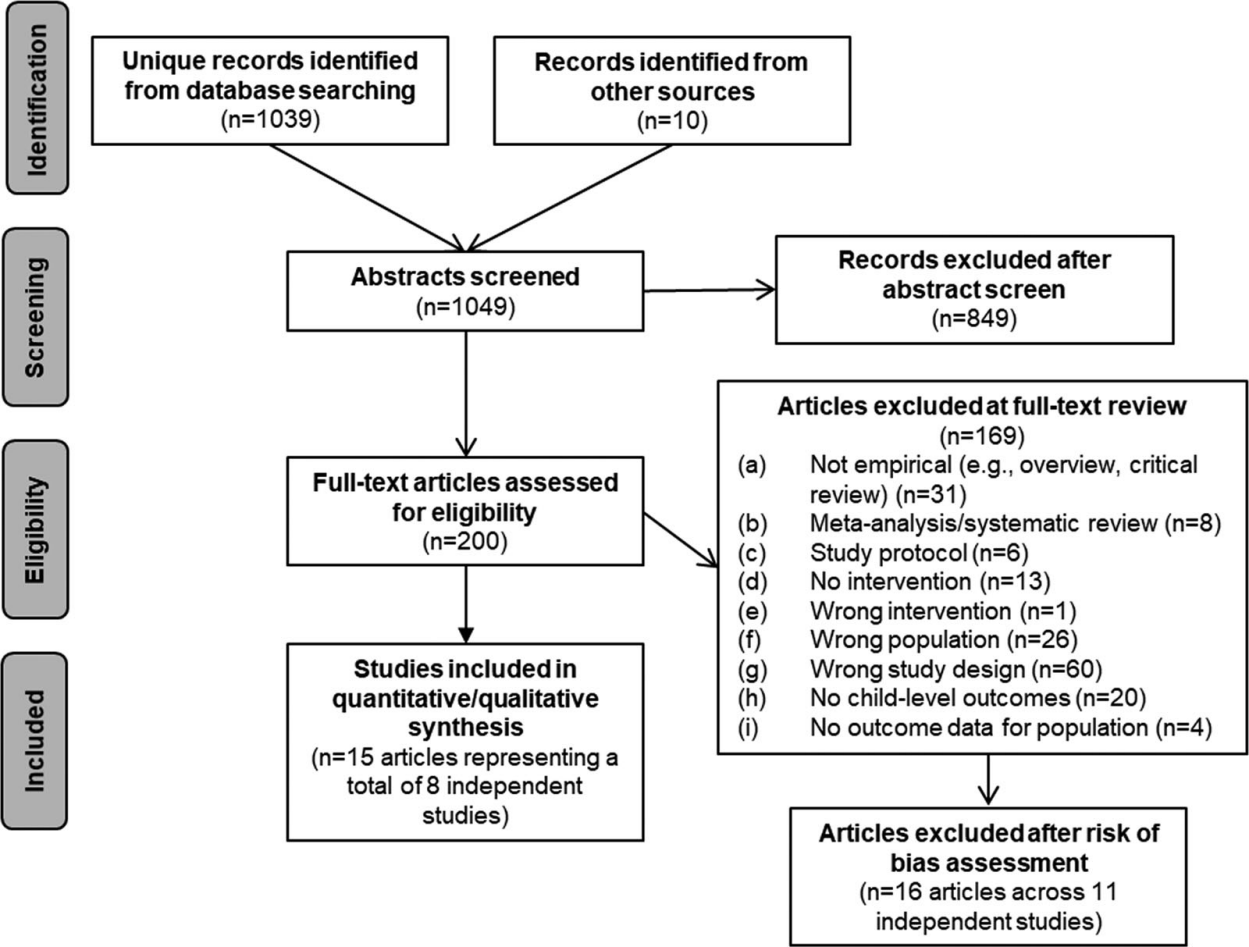
PRISMA flow diagram of study identification and selection. PRISMA, Preferred Reporting Items for Systematic Reviews and Meta‐Analyses

As illustrated in Figure 1, our search identified 1,049 unique titles, all of which underwent abstract screening. Of these, 200 articles were retrieved for closer analysis of the full‐text based on the information included in the title and abstract. Subsequently, we excluded 169 full‐text reports that did not meet inclusion criteria leaving 31 articles for which we completed a risk of bias assessment. Finally, 16 articles (across 10 independent studies) studies were excluded due to coder assessed high risk of bias. This left 15 articles across eight independent studies in our final sample.

#### 6.1.2 Inter‐rater reliability

Two trained researchers (N. E. L. and J. B.) independently assessed the 169 reports for inclusion or exclusion. A second member of the team (N. E. L. and J. B.; opposite reports than those originally reviewed) then reviewed all inclusion and exclusion decisions. Disagreements were resolved via discussion and on occasion, consultant by a third team member (C. C. and V. L. F.‐H.). Subsequently, three trained reviewers (N. E. L., J. B., and C. C.) extracted the relevant data from each included article into the evidence tables. A second member of the team (N. E. L. and J. B.; opposite reports than those originally reviewed) reviewed all data abstractions for completeness and accuracy. Here again coding disagreements were resolved via discussion and consensus and on occasion, consultation of a third team member (V. L. F.‐H.).

#### 6.1.3 Excluded studies: Full‐text screening

Following our published protocol, we excluded a total of 169 reports at the full‐text level of screening. References for the studies excluded at the full text level of screening (with rationale for each exclusion decision) are provided in the references section. Many articles were excluded for multiple reasons; in the PRISMA (Preferred Reporting Items for Systematic Reviews and Meta‐Analyses) flow diagram and in the excluded references list, we report the first relevant reason in our a priori hierarchy (no new data—such as an overview, critical review or editorial; meta‐analysis/systematic review of intervention studies; study protocol; no intervention; wrong intervention; wrong population; wrong study design; no child‐level outcomes; no outcome data for population; high risk of bias). Below we describe each of these exclusion reasons in turn.

##### 6.1.3.1 No data/not original research

We excluded 31 reports because they did not present data or original research; that is, the reports were critical reviews (or another type of unsystematic review), opinions, commentaries, or editorials/letters to the editor.

##### 6.1.3.2 Meta‐analysis/systematic review

During the searches, we kept eight articles which corresponded to systematic reviews literature reviews and/or meta‐analyses related to interventions to promote well‐being following parent and/or child IPV exposure. These eight manuscripts were initially retained to examine reference lists, but eventually excluded once reference lists were checked.

##### 6.1.3.3 Study protocol

We excluded six study protocols which described study methodology but did not present outcome data. Five protocols did not meet inclusion criteria due to various reasons (e.g., wrong intervention, wrong population, wrong study design; Catherine et al., 2016; Loeffen, Lo Fo Wong, Wester, Laurant, & Lagro‐Janssen, 2011; Skerfving, Johansson, & Elgán, 2014; Tarzia et al., 2016; van Rosmalen‐Nooijens, Prins, Vergeer, Wong, & Lagro‐Janssen, 2013). The sixth protocol (Overbeek, de Schipper, Lamers‐Winkelman, & Schuengel, 2012) was referenced during the risk of bias classification but was ultimately excluded in favor of the two published studies stemming from the evaluation described (Overbeek, de Schipper, Lamers‐Winkelman, & Schuengel, 2013; Overbeek, de Schipper, Willemen, Lamers‐Winkelman, & Schuengel, 2017).

##### 6.1.3.4 No intervention

Thirteen reports were excluded because they did not involve an intervention; these studies were predominately etiological in nature or focused on outcomes following IPV exposure.

##### 6.1.3.5 Wrong intervention

One study was excluded because the intervention evaluated was not psychosocial in nature. In this case, Fredland et al. (2014) conducted an observational study examining the impact of an abused mother's decision to seek safe shelter or a protection order on maternal and child functioning.

##### 6.1.3.6 Wrong population

Twenty‐six reports were excluded because they included children broadly defined as “at risk” for IPV exposure (e.g., McWhirter, 2008), or children exposed to other forms of violence (e.g., organized violence, Rousseau, Benoit, Lacroix, & Gauthier, 2009). Other reports excluded for this reason examined the impact of prevention programming on general populations of children (e.g., Dahle & Archbold, 2014).

##### 6.1.3.7 Wrong study design

Sixty reports were excluded because they did not satisfy the study design characteristics specified in the protocol. Specifically, of the 60 reports, 23 were excluded because they were a program description or provided qualitative data only. For example, Behan (2013) provided qualitative data on families engaged with Spokane Safe Start Project in Spokane, Washington, a program designed for children exposed to IPV. Similarly, Ellison (2014) described the First Steps Domestic Violence Program, a program designed to address the mental health needs of infants and toddlers entering a domestic violence shelter. Another 37 reports were excluded because they did not include a comparison group. For example, Grip et al. (2012) employed a repeated measures design to evaluate the impact on behavioral functioning of a community‐based program for children exposed to IPV. Similarly, Herschell et al. (2017) examined preschool child behavior and mental health symptomatology before and after receiving Parent‐Child Interaction Therapy (PCIT).

##### 6.1.3.8 No child‐level outcomes

Twenty reports were excluded because they did not present child‐level outcomes. Studies excluded for this reason typically included outcomes at the level of the victimized parent (e.g., parent depression; Graham‐Bermann & Miller‐Graff, 2015), or, sometimes, at the level of the parent‐child relationships (e.g., parenting behaviors, Howell, Miller, Lilly, et al., 2015; parenting stress, McConnell, Barnard, & Taylor, 2017).

##### 6.1.3.9 No outcome data for population

Four studies did not report results for children who have been exposed to IPV (at least, but not limited to). For example, Raden (1998) examined the impact of Head Start involvement on children's adaptation of violence‐related stress; results were presented for the entire sample, not stratified by youth exposed to (but not limited to) IPV.

#### 6.1.4 Excluded studies: Risk of bias

The methodological quality of each publication included after full‐text review was evaluated for risk of bias. We subsequently excluded 16 reports (across 11 independent studies) due to ratings of high risk of bias. Risk of bias assessment for RCTs and observational studies deemed to have high risk of bias are shown in Appendix B in Tables 1 and 2, respectively.

We excluded 15 reports for five or more reasons, such as selection bias (e.g., poor randomization and lack of allocation concealment for trials, failure to control for confounding factors for observational studies), performance bias (e.g., not controlling for concurrently occurring or unintended interventions), attrition bias (e.g., substantial loss to follow‐up of >20% or differential loss to follow‐up of >15% without appropriate handling of missing data), detection bias (e.g., in outcome assessment), and poor treatment fidelity.

After consultation with a third rater outside the study team with expertize in evaluation of bias (Dr. Meera Viswanathan), we eliminated an additional study (Pernebo, Fridell, & Almqvist, 2018) with a high degree of unmeasurable selection bias and confounding. In this study, children were recruited from a community‐based agency (arm 1) and a child and adolescent psychiatry unit (arm 2). The authors reported significant group differences between the arms (e.g., age, contact with father, anxiety symptomatology, posttraumatic stress symptomatology, sexual concerns), but did not control for these baseline differences in analyses.

### 6.2 Risk of bias in included studies

**Figure 2:**
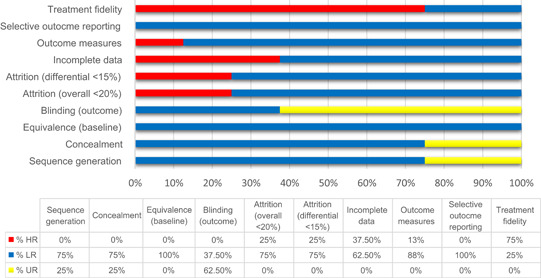
Risk of bias in included studies. Each of the 10 evaluated criteria were assigned one of three ratings: HR; LR, and UR. For the purposes of this graphic, conservative ratings were used—ratings of HR were given for some categories that had mixed ratings. See Appendix B for details. HR, high risk; LR, low risk; UR, unclear risk

Figure 2 summarizes the eight studies in our review deemed to be at overall moderate and low risk of bias. See Table 3 in Appendix B for a detailed risk of bias assessment for each included study.

### 6.3 Synthesis of results

Table 4 outlines characteristics of studies included in the review. Table 5 presents the types of IPV experienced by the parent and the nature of the child s exposure. Table 6 outlines the characteristics of study interventions and arms included in the review. Tables and figures outlining strength of intervention effects can be found in Appendix C.

#### 6.3.1 General Characteristics of Studies

Table 4 in Appendix C presents characteristics of the eight independent studies (15 articles) included in the review. All studies were published in peer‐reviewed journals. The first study that met inclusion criteria was published in 2001. The majority of studies were conducted in the United States (*n *= 6 independent studies, 75%), with others carried out in Western Europe (the Netherlands; *n *= 1, 12.5%) and Asia (India; *n *= 1, 12.5%). The total baseline sample sizes varied, from 36 (Jouriles et al., 2011) to 58 (McFarlane et al., 2005a). The age range of target children also varied; two studies examined outcomes across the entire span of childhood (age 18 months to 18 years in McFarlane et al., 2005a; age 0–16 years in Satyanarayana et al., 2016); five studies examined outcomes in children in a 6–8 years age span (e.g., age 4–9 years in Jouriles et al., 2009; age 7–14 years in Cohen et al., 2011); and one study focused on outcomes in children of a more narrow age band (age 3–5 years; Liberman et al., 2005).

Three included studies recruited general populations of parents and/or children who had been exposed to IPV, without stated inclusion criteria around parent or child symptomatology or functioning. Four studies had more explicit inclusion requirements, specifically: children with IPV‐related PTSD symptoms (Cohen et al., 2011), clinical levels of child behavior problems (Jouriles et al., 2011; Jouriles et al., 2009), concerning child behavior or parenting (Lieberman et al., 2005), and fathers with alcohol dependence syndrome (Satyanarayana et al., 2016). The most common exclusion reason (reported by six studies) was parent and/or child serious mental illness (e.g., psychosis).

Of note, studies varied widely regarding the nature of IPV experienced by parents and witnessed and/or heard by children. These variations are outlined in Table 5 in Appendix C and also described below.

##### 6.3.1.1 Exposure

Most included studies inferred exposure from either the mother's report of her own victimization (Cohen et al., 2011; Jouriles et al., 2009, 2001; McFarlane et al., 2005a; Overbeek et al., 2013) or the father's IPV perpetration (Satyanarayana et al., 2016). Two studies had more explicit definitions around exposure (Lieberman et al., 2005; McWhirter, 2011).

##### 6.3.1.2 Types of IPV

The types of IPV experienced or perpetrated by parents varied widely across included studies. Two studies (Jouriles et al., 2009, 2001) recruited mothers who had experienced at least physical IPV; two studies recruited mothers (McWhirter, 2011) or a parent (Overbeek et al., 2013) who had experienced physical or psychological IPV; one study examined outcomes in children with a mother who had experienced physical or sexual IPV (McFarlane et al., 2005a); and one study (Satyanarayana et al., 2016) recruited fathers who reported physical, psychological, or sexual IPV perpetration. Two studies (Cohen et al., 2011; Lieberman et al., 2005) did not explicate specific types of IPV experienced.

##### 6.3.1.3 Victim‐perpetrator relationship

All but one (Overbeek et al., 2013) included study focused on outcomes of children with a victimized mother. Of these seven studies, two explicitly noted the perpetrator must be a husband, two focused on mothers with male partners, and three were broader, referencing the mother's “partner.” Overbeek et al. (2013) described the most inclusive victim‐perpetrator relationship: victimized parent (of either sex) and perpetrating partner (of either sex).

#### 6.3.2 Characteristics of interventions evaluated

Table 6 in Appendix C presents characteristics of the interventions evaluated in the eight independent studies (15 articles) included in the review. There was great heterogeneity in intervention types and comparators, the intervention recipient or modality, and the intervention delivery setting. Intervention types ranged from cognitive‐behavioral therapy for the child, mother, and/or perpetrating father (Cohen et al., 2011; McWhirter, 2011; Overbeek et al., 2013; Satyanarayana et al., 2016), to intensive parent training (Jouriles et al., 2009, 2001), CPP (Lieberman et al., 2005), and nurse case management (CM; McFarlane et al., 2005). Three studies compared an active intervention to either an attention control (Jouriles et al., 2009, 2001) or inactive control (McFarlane et al., 2005a), whereas five studies (Cohen et al., 2011; Liberman et al., 2005; McWhirter, 2011; Overbeek et al., 2013; Satyanarayana et al., 2016) were comparative effectiveness studies that compared two active interventions.

Included studies examined the impact of interventions delivered to the mother only (Jouriles et al., 2009, 2001; McFarlane et al., 2005a), mother and child together (here defined as any number of joint sessions; Cohen et al., 2011; Lieberman et al., 2005, McWhirter, 2011), mother and child separately (Overbeek et al., 2013), and perpetrating father only (Satyanarayana et al., 2016). Jouriles et al.'s (2009, 2001) intensive parenting intervention was delivered in the parent's home, whereas other interventions were delivered in outpatient settings, such as community centers and clinics (Cohen et al., 2011; Lieberman et al., 2005; McFarlane et al., 2005a; Overbeek et al., 2013), homeless family shelters (McWhirter, 2011), and inpatient psychiatric clinics (Satyanarayana et al., 2016).

Only one study reported tracking adverse events. In this study, Cohen et al. (2011) found that families participating in TF‐CBT reported fewer instances of serious physical IPV, reportable episodes of child abuse, child self‐injury, and other serious problems requiring psychiatric hospitalization than did participants in the Child Centered Therapy Group (3.65% vs. 41.25%).

#### 6.3.3 Outcome: Overall problems

Two trials examined overall (or total) problems among the full study sample, both using the Child Behavior Checklist (CBCL; Achenbach, 1991). One study reported between group changes immediately posttreatment (Lieberman et al., 2005) and the other reported total problem outcomes at 1‐ and 3‐month posttreatment (Satyanarayana et al., 2016). Table 7 in Appendix C shows the effect sizes for each study that included overall problem outcomes. Findings were not pooled due to differences in (a) interventions; (b) comparison interventions (“comparator); and (c) timing of outcomes.

Lieberman et al. (2015) found that that participants in a CPP group had greater decreases in total behavioral problem scores at posttreatment than participants in a (CM) plus treatment as usual (TAU) in the community group (SMD = −0.56, 95% CI [−1.06 to −0.06]). Satyanarayana et al. (2016) found no significant differences in total problem behavior at 1‐ and 3‐months posttreatment between participants in the integrated cognitive behavioral intervention (ICBI) group and the TAU group.

#### 6.3.4 Outcome: Externalizing behavior

Five trials used the CBCL to examine externalizing behavior outcomes among the full study samples. One trial compared TF‐CBT with child‐centered therapy (CCT) and reported externalizing behavior immediately posttreatment only (Cohen et al., 2011). Two trials compared externalizing behavior immediately posttreatment and at 6 months posttreatment, one testing CPP versus CM plus individual therapy (Lieberman et al., 2005) and the other comparing a specific factors intervention versus a nonspecific factors intervention (Overbeek et al., 2013). The other two trials each compared an intensive services intervention versus an attention control condition immediately posttreatment, and at 4‐ and 8‐month follow‐ups, permitting meta‐analyses of findings across these two studies at each of these time points (Jouriles et al., 2001; Jouriles et al., 2009). Findings were not pooled for other studies because of variation in intervention types and comparators (some employed a control group whereas others were comparative effectiveness studies that compared two active interventions). In addition, one of the trials also reported three other externalizing measures, the Eyberg Child Behavior Inventory (ECBI; Eyberg & Ross, 1978) (immediately and at 12 months posttreatment), oppositional behavior as coded from observational data using a code developed by Hetherington and Clingempeel (1986) (immediately and at 16 months posttreatment), and the Psychopathy Screening Device (PSD; Frick, O'Brien, Wootton, & McBurnett, 1994) (immediately posttreatment only) (ECBI and observed oppositional behavior in Jouriles et al., 2009; PSD in McDonald, Jouriles, & Minze, 2011).

**Figure 3:**
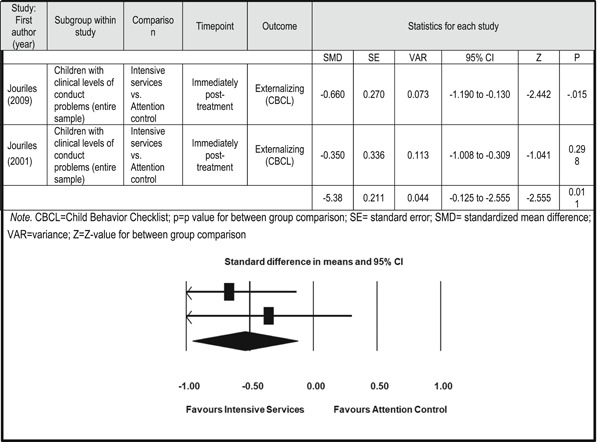
CBCL externalizing score effect sizes immediately posttreatment: meta‐analysis of two studies. CBCL, Child Behavior Checklist

**Figure 4:**
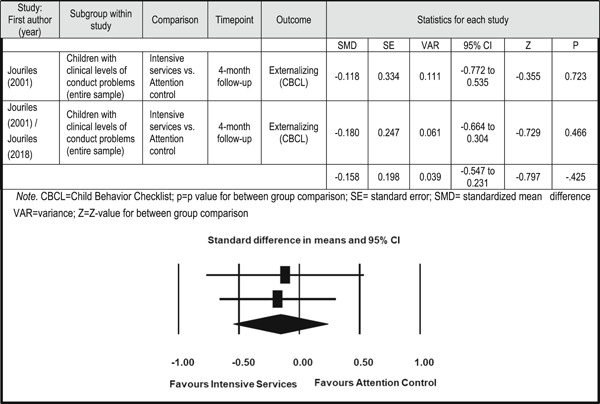
CBCL externalizing score effect sizes 4‐month posttreatment: meta‐analysis of two studies. CBCL, Child Behavior Checklist

**Figure 5:**
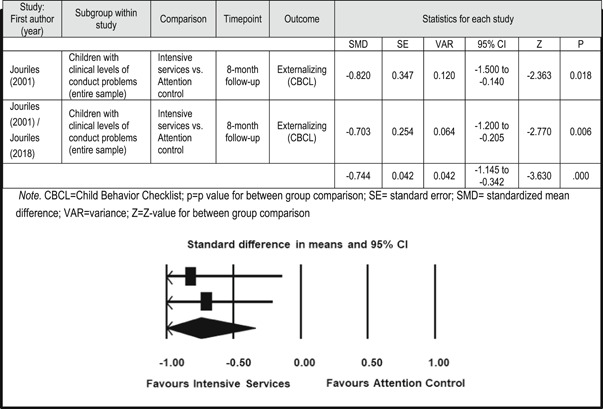
CBCL externalizing score effect sizes 8‐month posttreatment: meta‐analysis of two studies. CBCL, Child Behavior Checklist

Table 8 in Appendix C shows the standardized effect sizes for each of these studies as well as the three aforementioned pooled estimates. Two studies found no significant differences in externalizing behavior scores: (a) immediately posttreatment comparing participants in a TF‐CBT and CCT group (Cohen et al., 2011), and (b) immediately or at 6‐month follow‐up comparing those in a specific factors intervention and a nonspecific factors intervention (Overbeek et al., 2013). A third study found that participants in a CPP group had greater decreases in CBCL externalizing scores immediately posttreatment and also at 6‐month follow‐up than participants in a CM plus individual therapy group (Lieberman et al., 2005). For the two studies testing intensive services versus an attention control group, the first found no significant group differences in CBCL‐measured externalizing behaviors immediately posttreatment or at 4‐month follow‐up, but did find that participants in an intensive services group had greater decreases in externalizing behaviors at 8‐ and 24‐month follow‐ups than an attention control group (Jouriles et al., 2011); whereas the second trial found significant differences immediately posttreatment and at 8‐, 16‐, and 20‐month follow‐ups but not at 4‐ or 12‐month follow‐ups (Jouriles et al., 2009). Pooling the findings from the two studies together, the meta‐analyses demonstrated homogeneity in effects (*I* ^2^ = 0.00 in each instance). Participants in the intensive services group had greater decreases in CBCL externalizing behavior scores than attention control group participants immediately posttreatment (SMD = −0.54, 95% CI [−0.95 to −0.13], two studies, *I* ^2^ = 0.00; Figure 3) and at 8‐month follow‐up (SMD = −0.74, 95% CI [−1.15 to −0.34], I^2^ = 0.00; Figure 5) but not significantly so at 4‐month follow‐up (SMD = −0.16, 95% CI [−0.55 to 0.23], two studies, *I* ^2^ = 0.00; Figure 4).

#### 6.3.5 Outcome: Internalizing behaviors

Three trials examined internalizing behavior among the full study samples. Two tested internalizing behaviors as assessed by the CBCL (one comparing an intensive services intervention vs. an attention control condition immediately posttreatment and at 4‐, 8‐, and 24‐month follow‐ups; Jouriles et al., 2001) and the other comparing a goal‐oriented intervention to an emotion‐focused intervention immediately posttreatment only (McWhirter, 2011), and the other trial used a visuographically emotional barometer to compare internalizing behavior between participants receiving a specific factors intervention compared with participants receiving a nonspecific factors intervention immediately posttreatment and at 6‐month follow‐up (Overbeek et al., 2013). Findings were not pooled because of variation in intervention types and comparators (one employed a control group whereas other two trials were comparative effectiveness studies that compared two active interventions).

Table 9 in Appendix C shows the standardized effect sizes for each of these studies. No significant differences were found in internalizing behaviors compared between participants in the goal‐oriented intervention and emotion‐focused intervention groups immediately posttreatment (McWhirter, 2011) or between who received a specific factors intervention compared with participants who received a nonspecific factors intervention immediately posttreatment and at 6‐month follow‐up (Overbeek et al., 2013). The third study found that intensive services group participants had significantly greater decreases in internalizing behavior scores immediately posttreatment and at 8‐month follow‐up than attention control group participants, but no significant between group differences at the 4‐ or 24‐month follow‐ups (Jouriles et al., 2001).

#### 6.3.6 Outcome: Posttraumatic stress symptomatology

Two trials examined posttraumatic stress among the full study samples. One study tested PTSD scores on the Diagnostic Classification of Mental Health and Developmental Disorders of Infancy and Early Childhood (DC 0–3; Zero to Three: National Center for Clinical Infant Programs, 1994) immediately posttreatment between participants who received CPP and those who received CM plus individual therapy (Lieberman et al., 2005). The other study examined scores on the Trauma Symptom Checklist for Children (TSCC)/for Young Children (TSCYC; Briere, 1997) immediately and at 6‐month follow‐up and TSCYC scores at 6‐month follow‐up between participants in a specific factors intervention and those in a nonspecific factors intervention (Overbeek et al., 2013). Findings were not pooled because of variation in intervention types and comparators.

Table 10 in Appendix C shows the standardized effect sizes for both of these studies. Participants in the CPP group had significantly greater decreases in posttraumatic stress symptomatology scores than those in the CM plus individual therapy group immediately posttreatment (Lieberman et al., 2005). The other study found no significant between‐group differences in TSCC scores immediately and at 6‐month follow‐up but did find that participants in the specific factors intervention had greater decreases in PTSD scores on the TSCYC at 6‐month follow‐up than those in the nonspecific factors intervention (Overbeek et al., 2013).

#### 6.3.7 Outcome: Other (anxiety, depression, cognitive ability, social relationships)

Three trials examined other child‐level outcomes among the full study samples. One study compared anxiety using the Screen for Child Anxiety Related Emotional Disorders (SCARED; Birmaher et al., 1997), a self‐report measure of non‐PTSD anxiety, cognitive ability using the Kaufman Brief Intelligence Test (KBIT; Kaufman & Kaufman, 1990), and depression using the Children's Depression Inventory (CDI; Kovacs, 1985) immediately posttreatment between participants who received TF‐CBT and those who received CCT (Cohen et al., 2011). Another trial compared depression using the CDI between participants receiving a specific factors intervention compared with participants receiving a nonspecific factors intervention immediately posttreatment and at 6‐month follow‐up (Overbeek et al., 2013). The third trial reported 24‐month follow‐up differences in mothers’ assessments of their child's happiness and social relationships using four items drawn from the Health Resources Inventory (Gesten, 1976) in participants who received an intensive services intervention versus those who received an attention control intervention (McDonald et al., 2006, part of Jouriles et al., 2001). Findings were not pooled because of variation in intervention types, compartors or outcomes.

Table 11 in Appendix C shows the standardized effect sizes for each of these studies. Immediately posttreatment, participants in the TF‐CBT group had significantly greater improvements in anxiety and cognitive ability scores than those in the CCT group, but depression scores did not significantly differ between these groups (Cohen et al., 2011). Depression scores immediately posttreatment and at 6‐month follow‐up also did not significantly differ between those in the specific factors intervention and those in the nonspecific factors intervention. The third study found no significant differences in child happiness/social relationships as reported by their mothers at 24‐month follow‐up between intensive services and attention control group participants (McDonald et al., 2006, part of Jouriles et al., 2001).

#### 6.3.8 Subgroup analyses

##### 6.3.8.1 Age of participant

One study (McFarlane et al., 2005a) examined whether the impact of a nurse CM intervention (compared to an inactive control group) differed between younger (ages 18 months to 5 years old) and older (ages 6–18 years old) children. Outcomes examined included total problem behavior, externalizing behavior, and internalizing behavior using the CBCL, measured at 6‐, 12‐, 18‐, and 24‐month follow‐up. As shown in Table 12 in Appendix C, no significant differences were reported for any outcome within or between age groups at any of the follow‐up time points.

##### 6.3.8.2 Number of traumatic life events

One study (Ghosh Ippen, Harris, Van Horn, & Lieberman, 2011; part of Lieberman et al., 2005) focused on the comparative effectiveness of CPP versus a CM plus individual therapy intervention examined two subgroups: youth with four or more (“high risk”) and youth with less than four lifetime (“low risk”) lifetime exposures to traumatic and stressful life events. Total problem behaviors (CBCL scores) were reported immediately and at 6‐month posttreatment, and PTSD and depression (each measured using the DC 0–3) were reported immediately posttreatment only. As shown in Table 13 in Appendix C, for each outcome, the CPP intervention was superior to the CM plus individual therapy intervention only among high risk, but not low risk, children.

#### 6.3.9 Metaregression

##### 6.3.9.1 Intervention recipient/modality

To answer our research question regarding whether the intervention recipient (“modality”) impacts efficacy or effectiveness of tested interventions, we intended to conduct a meta‐regression. We deemed this potentially possible for only one outcome, with five studies reporting changes in CBCL‐measured externalizing behaviors immediately postintervention. As shown in Table 14 in Appendix C, two studies targeted families together—with the dyad as the unit of treatment, two studies targeted the nonoffending parent only (in both studies, the child's mother), and one study examined outcomes of an intervention delivered concurrently but separately to the nonoffending parent and child. Upon evaluation of our data, however, we did not believe it was appropriate to conduct a formal quantitative meta‐regression because of heterogeneity in study characteristics such as the nature of the comparators. For example, the interventions targeting the nonoffending parent both compared an intervention to an attention control, whereas the other three interventions each had an active comparator. Instead, we present here the pooled findings of the studies grouped by the targets of the intervention (e.g., families together, nonoffending parent only, and nonoffending parent separately from child), we found that studies targeting the nonoffending parent (mother) had the highest pooled effect size (SMD = −0.38, 95% CI [−0.62 to −0.14], *p *< .01), followed by those that targeted the family together (SMD = −0.25, 95% CI [−0.53 to 0.04], *p *= *ns*) and, finally, the single study that targeted parent and child, separately (SMD = −0.08, 95% CI [−0.45 to 0.26], *p* = *ns*). Upon further inspection of the nature of the comparators, however, we determined that the interventions targeting the nonoffending parent (mother) both compared the intervention to an attention control, whereas the other three interventions (which targeted parents together and nonoffending and child separately) each had an active comparator. It is not unsurprising that efficacy studies would have larger effect sizes than comparative effectiveness studies, where both group participants receive an active intervention. Thus, these findings should be interpreted with great caution.

##### 6.3.9.2 Setting

Another research question sought to determine whether the setting of the intervention impacted the efficacy or effectiveness of the tested interventions. Like modality described in the prior section, we had intended to conduct another meta‐regression for the five studies that reported changes in CBCL‐measured externalizing behaviors immediately postintervention, but, again, heterogeneity in study characteristics precluded us doing this quantitively. Instead, we present the pooled findings of the studies grouped by settings of the intervention. As shown in Table 15, Appendix C, two interventions were delivered family home and the other three were delivered an outpatient clinical setting. Pooled findings indicated that studies conducted in the home had a moderately significant pooled effect size (SMD = −0.38, 95% CI [−0.62 to −0.14], *p *< .01), and the effect size of studies conducted in an outpatient setting did not reach statistical significance (SMD = −0.25, 95% CI [−0.53 to 0.04], *p *= *ns*).

These findings should be interpreted with great caution because of heterogeneity in study characteristics (e.g., nature of the comparators). The interventions conducted in the home setting were efficacy studies, whereas the interventions conducted in outpatient settings each employed an active control group. Again, it is expected that efficacy studies would have larger effect sizes than effectiveness studies.

#### 6.3.10 Publication bias analysis

**Figure 6:**
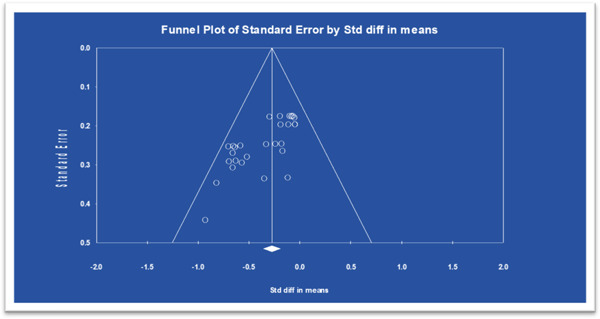
Assessment of publication bias for studies that reported externalizing behavior outcomes

We conducted analyses to determine whether the included studies adequately represent the whole population of completed studies, and not just those that have been published in the literature. There is evidence that studies reporting larger effect sizes are more likely to get published than those showing small or no effects of a tested intervention (Rothstein, Sutton, & Borenstein, 2006). To examine the potential for publication bias, we constructed a funnel plot that illustrates the effect sizes by standard errors of the effects. We chose to examine publication bias for the studies that tested externalizing outcomes, which was the outcome most widely reported by the studies included in our meta‐analysis. As illustrated in Figure 6 in Appendix C, the funnel plot does not illustrate evidence of publication bias, with the effect sizes (standardized difference in means) plotted against the standard errors indicating a normally distributed, symmetrical distribution of points under the funnel. Individual study points are distributed around the main effect, with all points falling under the funnel. However, since the evaluation of funnel plots can be subjective, we conducted additional statistical measures of publication bias, specifically, Duval and Tweedie's trim‐and‐fill analysis. This additional analysis did not suggest trimming any studies. We note that because these analyses of studies included in our review are only from published studies, however, we cannot be certain that no publication bias exists.

### 6.4 Sensitivity analysis

Since the present meta‐analysis involved a wide range of decisions, we conducted sensitivity analysis to test the robustness of these decisions (Higgins & Green, 2011). Specifically, we ran sensitivity analysis for the pre‐post correlations (i.e., covariance) assumed to be .70. We re‐ran the analysis using a correlation equal to 0.50. As expected there was no change in the effect sizes and no relevant difference in standard errors.

## 7 DISCUSSION

### 7.1 Summary of main results

The objective of this review was to synthesize the evidence on the impact of psychosocial interventions on well‐being among children exposed to IPV. We also sought to examine whether impact was related to intervention modality (e.g., individual, family‐based) or setting (e.g., home, outpatient clinic).

Below we discuss the *main findings* from our systematic review and meta‐analysis. When applicable, we also discuss, qualitatively, findings from key studies ultimately excluded due to high risk of bias.

Although studies were searched from the earliest search date available to April 2018, the first study that met inclusion criteria was published in 2001, with half of the primary study papers published since 2011. We identified eight RCTs, with a total of 924 participants, reporting results on the impact of a range of interventions designed to promote well‐being among children exposed to IPV. However, the great clinical and methodological variability of included studies largely precluded pooling of trials. Specifically, there was a high degree of heterogeneity regarding differences in interventions employed, comparators (some studies employed a control group whereas others were comparative effectiveness studies that examined two active interventions), outcomes examined, and timing of outcomes (i.e., length of follow‐up period). Therefore, it is unclear the extent to which psychosocial interventions promote well‐being among children exposed to IPV, and under what circumstances, such as the modality and setting. Of note, only one study reported tracking adverse events. This is surprising, given the high‐risk nature of study populations and the established finding that IPV tends to co‐occur with child maltreatment and other risk behaviors such as substance use (e.g., Chan, 2011; Dube, Anda, Felitti, Edwards, & Williamson, 2002).

#### 7.1.1 Externalizing behavior

We were only able to pool results from two studies, both of which examined the impact of Project Support (intensive services intervention focused on parent training and emotional support) compared to an attention control on child externalizing behavior—among youth referred for clinical levels of conduct problems. This meta‐analysis found that participants reported greater decreases in their child's externalizing behavior immediately posttreatment and at an 8‐month follow‐up, but not at a 4‐month follow‐up. One of these pooled studies (Jouriles et al., 2001) also found decreases in internalizing behavior in the Project Support group (compared to the attention control) immediately posttreatment and at the 8‐month follow‐up, but not at the 4‐ or 24‐month follow‐up. Mixed results regarding impacts on externalizing behaviors were found in comparative effectiveness trials, with only one of three trials finding significant group differences.

#### 7.1.2 Other outcomes

We could not pool trials examining other outcomes due to the great heterogeneity mentioned above. Only one study examined impacts on non‐posttraumatic stress anxiety and cognitive ability, finding greater positive impacts for a TF‐CBT group compared to a CTT group (Cohen et al., 2011). Qualitatively, there are mixed findings regarding intervention impacts on internalizing behaviors and posttraumatic stress symptomatology, perhaps due to the variability in the types of participants included across trials. For example, Ghosh Ippen et al. (2011, part of Lieberman et al., 2005) found that CPP was more efficacious than CM and individual therapy in reducing posttraumatic stress symptomatology and depression for high risk (higher number of exposures to traumatic life events) but not low risk (lower number of exposures to traumatic life events) children. This finding is consistent with two studies that were ultimately excluded due to high risk of bias. First, Foshee et al. (2015) found that a mother‐child self‐directed intervention (mailed booklets of dating abuse prevention information and interactive activities) was effective in reducing dating violence victimization for adolescents with higher, but not lower, levels of exposure to domestic violence. Second, Pernebo et al. (2018) found that compared to a group psychoeducational intervention, a group psychotherapeutic intervention (parallel groups children and parents) for was effective at reducing child mental health problems, particularly for those children with higher levels of pretreatment trauma symptomatology.

#### 7.1.3 Modality

With regard to modality, pooled findings indicate that studies targeting the nonoffending parent (mother) had the highest pooled effect size (SMD = −0.38, 95% CI [−0.62 to −0.14], *p *< .01), followed by those that targeted the family together (SMD = −0.25, 95% CI [−0.53 to 0.04], *p *= *ns*) and, finally, the single study that targeted parent and child, separately (SMD = −0.08, 95% CI [−0.45 to 0.26], *p *= *ns*). These findings should be interpreted, however, with great caution because of heterogeneity in study characteristics such as the nature of the comparators. For example, the two interventions targeting the nonoffending parent both compared an intervention to an attention control, whereas the other three interventions each had an active comparator.

Interestingly, no studies removed due to high risk of bias evaluated interventions delivered to the parent only. Most studies removed due to risk of bias evaluated family‐based interventions in which parent‐child pairs received separate but parallel (Graham‐Bermann et al., 2007; Graham‐Bermann, Miller‐Graff, Howell, & Grogan‐Kaylor, 2015; Pernebo et al., 2018; Sullivan, Bybee, & Allen, 2002) or joint (Foshee et al., 2015; Waldman‐Levi & Weintraub, 2009; Smith, Belton, Barnard, Fisher, & Taylor, 2015) programming. Three additional studies evaluated interventions delivered to children only (Kot, 1995; Smith & Landreth, 2003; Tyndall‐Lind, Landreth, & Giordano, 2001; Wagar & Rodway, 1995). It is unclear whether this is chance, speaks to broader challenges with minimizing selection bias with parent‐child programming, challenges with evaluating an active comparator (versus an attention control, as was used in the two pooled studies examining parent‐only interventions), or some other factor entirely. Importantly, the broader literature as well as excluded studies point to the potential value of family‐based treatment. For example, Graham‐Bermann et al. (2007) found that a child‐plus‐mother intervention (Kids’ Club and MEP) was more effective at reducing children's externalizing and internalizing behavior problems compared to a child‐only intervention (Kids’ Club) and a no‐treatment control.

#### 7.1.4 Setting

With regard to setting, pooled findings indicated that studies conducted in the home had a larger (and significant) pooled effect size (SMD = −0.38, 95% CI [−0.62 to −0.14], *p *< .01) as compared with those conducted in an outpatient setting (SMD = −0.25, 95% CI [−0.53 to 0.04], *p *= *ns*). Like the findings focused on modality, these findings focused on setting differences should be interpreted with great caution because of heterogeneity in study characteristics (e.g., nature of the comparators). The interventions conducted in the home setting were efficacy studies, whereas the interventions conducted in outpatient settings each employed an active control group. It is expected that efficacy studies would have larger effect sizes than effectiveness studies.

Given the inconclusive findings from our review, below we discuss three general conclusions and provide recommendations for future research to build the evidence base.

First, the evidence base remains underdeveloped and characterized by some breadth at the sake of depth. This breath became evident during our full‐text review and systematic application of inclusion and exclusion criteria. For example:

- Sixty reports were excluded because they did not meet study design requirements as specified in the protocol. Twenty‐three of these articles provided a program description or presented qualitative data only, and another 37 were excluded because they did not include a comparison group. This suggests there is great interest in developing, describing, and evaluating programs for children exposed to IPV. Several recent observational studies show promise and warrant more rigorous evaluation (e.g., Herschell et al., 2017; Lacasa et al., 2016), as do practice‐based programs described by many of the excluded qualitative reports. Practice‐based programs (or those developed in the field and not in the “ivory tower”) have the advantage of uptake in practice and some degree of demonstrated acceptability by interventionists and the contexts (e.g., shelters) in which they are implemented. Funding opportunities that require partnership between researchers and practitioners may help advance the evidence‐based on practice‐based programs. Such calls for rigorous evaluation of practice‐based programs have been made in the field of violence prevention (Centers for Disease Control and Prevention, 2016).
- Twenty reports were excluded because they did not present child‐level outcomes. Studies excluded for this reason met other study design requirements (e.g., RCT or quasiexperimental design with a comparison group), but typically examined outcomes at the level of the victimized parent. A recent meta‐analysis examining the effectiveness of interventions to promote well‐being among survivors of IPV found that short‐term interventions (typically cognitive‐behavioral in nature) were effective in reducing PTSD symptomatology, depression and general distress, and increasing self‐esteem and general life functioning—particularly in studies that employed a control group (Arroyo, Lundahl, Butters, Vanderloo, & Wood, 2017). Given the large literature demonstrating the negative impact of parent psychopathology on child functioning (e.g., Cummings & Davies, 1994), addition of child‐level measures to parent‐focused evaluations would be a contribution to the evidence base and with relatively minimal resources.

Similar to Howarth et al. (2016), we suggest a “pause” in the development of new psychosocial interventions unless they address a clear gap in service delivery that *cannot* be addressed by adapting components of existing theoretically driven programs. For example, Herschell et al. (2017) conducted a feasibility trial of PCIT in a domestic violence shelter; although results indicated that PCIT had a positive impact on child behavior and mental health symptomatology, only 42% of families completed the full course of treatment. These results suggest that PCIT holds promise, but likely needs to be adapted from the standard protocol (weekly, 1‐hr sessions) to meet the needs of families in a shelter setting. Similarly, as noted earlier, we found very few replication studies—our meta‐analyses of externalizing outcomes were in fact limited to a single replication study conducted by the same team as the original study (Jouriles et al., 2009, 2001). Paired with the fact that four (50%) studies in our review had fewer than 100 total participants, well‐powered replication studies conducted by independent research teams will undoubtedly help move the field forward.

Second, although 19 independent studies met our full‐text review inclusion criteria, 11 of these studies were ultimately excluded due to high risk of bias. Our decision to examine only studies assessed to be at low or moderate risk of bias was conservative, built from the concerns expressed by authors of prior reviews (e.g., Hackett et al., 2016; Howarth et al., 2016; Rizo et al., 2011) that conclusions around “what works” have been drawn from a literature dominated by studies with methodological limitations. Trials evaluating the widely disseminated Kids’ Club, Pre Kids’ Club and MEP (Graham‐Bermann et al., 2007, 2015; Howell, Miller, Lilly, & Graham‐Bermann, 2013; Miller, Howell, Hunter, & Graham‐Bermann, 2012) were ultimately removed due to high selection bias (also coded as high risk of bias in Howarth et al., 2016). Given these programs are theory‐driven, rated as “promising” by the California Evidence‐Based Clearinghouse for Child Welfare (CEBC; 2016) and have demonstrated uptake the field, including internationally (used in Sweden and Canada; CEBC, 2016), we recommend that these programs undergo rigorous evaluation by independent research teams.

Third, consistent with the larger literature (Latzman et al., 2017) included studies generally failed to acknowledge the varied ways in which children come to know about their parent's IPV victimization (exposure) or consider the full range of the types of IPV to which children can be exposed. Most studies inferred exposure from either the mother's report of her own victimization or child residence in a domestic violence shelter. As noted by Latzman et al. (2017), a growing body of research indicates that the type of exposure may be a key element helping to explain differential outcomes, with children most negatively impacted when they are directly involved in the violence (e.g., Jarvis, Gordon, & Novaco, 2005; Jouriles, Rosenfield, McDonald, & Mueller, 2014). Similarly, most included studies—if the type of IPV was explicated—focused on mother's report of physical IPV victimization, or physical or sexual/psychological IPV victimization. Only one study (Satyanarayana et al., 2016) included fathers who reported physical, psychological, and/or sexual IPV perpetration. Thus, the scope of this review is ultimately narrower than the title implies; this review speaks to the impact of interventions on well‐being among children *whose mother reported physical (and possibly, sexual, or psychological) IPV victimization*. Future research should consider assessing the full range of ways in which children are exposed (direct involvement, direct eyewitness, indirect exposure) to multiple types of IPV (physical, sexual, and psychological aggression, and stalking).

### 7.2 Overall completeness and applicability of evidence

The majority of evidence included in this review was generated in the last 10–15 years. Most studies were conducted in the United States, with one study each from the Netherlands and India. Thus, findings from our review are only applicable to these countries and additional evidence from outside of the United States is needed to better inform practice and policy in international contexts.

### 7.3 Quality of the evidence

As noted, we only included studies in this review that were rated to have a low or moderate risk of bias—with only one study assessed as having a low risk of bias. All included studies evidenced limited to no selection, attrition or reporting bias. Detection bias was rated as either “low” or “unclear” for all included studies.

### 7.4 Limitations and potential biases in the review process

Our search as limited to 10 electronic databases and scans of relevant review articles. Thus, it is possible that we may have missed studies. As noted, 50% of the included studies reported results based on samples with <100 participants. The small samples sizes could impose limitations on the ability to detect effects of interventions.

### 7.5 Agreements and disagreements with other studies or reviews

The review that most closely matches with the present review is that of Howarth et al. (2016), who conducted a mixed‐methods review of findings from studies that tested interventions to improve outcomes for children exposed to domestic violence and abuse published up through April 2013. The authors concluded that the evidence base, even from this comprehensive examination of evidence, lacked empirical data testing the clinical effectiveness, cost effectiveness, and acceptability of these interventions. Our review is an advancement on this work, including five more years of published literature and providing information on the most internally valid studies available. Nonetheless, the evidence base appears to have made limited advancements over the last five years, with our conclusions consistent with that of Howarth et al. (2016): more rigorous evaluations need to be conducted in order to draw conclusions. We also suggest that in addition to increased rigor in evaluation design, researchers assess the nature of child exposure and the multiple subtypes of IPV to which children can be exposed.

## 8 AUTHORS' CONCLUSIONS

### 8.1 Implications for practice and policy

The findings from this systematic review indicate that it is largely unclear the extent to which psychosocial interventions promote well‐being among children exposed to IPV, and under what circumstances.

### 8.2 Implications for research

The main research need that emerges from this review is more rigorous evaluation (e.g., RCTs or quasiexperimental trials with strong internal validity) of psychosocial interventions to promote well‐being among children exposed to IPV. There are simply too few comparable studies (i.e., in interventions, comparators, outcomes) conducted to draw conclusions at this point in time.

Given the inconclusive findings from our review, below we outline three general conclusions and recommendations for future research to build the evidence base.

First, the evidence base remains underdeveloped and characterized by some breadth at the sake of depth. This breath became evident during our full‐text review and systematic application of inclusion and exclusion criteria. For example:

- Twenty‐three reports provided a program description or presented qualitative data only, and another 37 evaluated programs but failed to include a comparison group. This suggests there is great interest in developing, describing, and evaluating programs for children exposed to IPV. Several recent observational studies show promise and warrant more rigorous evaluation (e.g., Herschell et al., 2017, Lacasa et al., 2016).
- Twenty reports were excluded because they did not present child‐level outcomes. Studies excluded for this reason typically examined outcomes at the level of the victimized parent. Given the large literature demonstrating the negative impact of parental psychopathology on child functioning (e.g., Cummings & Davies, 1994), addition of child‐level measures to parent‐focused evaluations would be a contribution to the evidence base and with relatively minimal resources.
- We found very few replication studies—our meta‐analyses of externalizing outcomes were in fact limited to a single replication study conducted by the same team as the original study (Jouriles et al., 2009, 2001). Paired with the fact that half of the studies in our review had fewer than 100 total participants, well‐powered replication studies conducted by independent research teams will undoubtedly help move the field forward.

Second, although 19 independent studies met our full‐text review inclusion criteria, 11 of these studies were ultimately excluded due to high risk of bias. Again, this highlights the breadth of programming, but unfortunately, lack of internal validity of a large proportion of existing work. We recommend—particularly for programs with uptake in the field (e.g., Kids' Club, Pre Kids' Club and MEP)—more rigorous evaluation by independent research teams. It is particularly important for researchers to design studies that minimize biases in sample definition and selection.

Third, consistent with the larger literature (Latzman et al., 2017) included studies generally failed to acknowledge the varied ways in which children come to know about their parent's IPV victimization (exposure) or consider the full range of the types of IPV to which children can be exposed. Future research should consider assessing the full range of ways in which children are exposed (direct involvement, direct eyewitness, indirect exposure) to multiple types of IPV (physical, sexual, and psychological aggression, and stalking).

## INFORMATION ABOUT THIS REVIEW

### REVIEW AUTHORS

**Lead review author**: The lead author is the person who develops and co‐ordinates the review team, discusses and assigns roles for individual members of the review team, liaises with the editorial base and takes responsibility for the on‐going updates of the review.

### ROLES AND RESPONSIBILITIES

Content: N. E. L. and C. C. Systematic review methods: N. E. L. and V. L. F.‐H. Statistical analysis: N. E. L. and V. L. F.‐H. Information retrieval: N. L., J. B., V. L. F.‐H.

The team was led by N. E. L., who has substantive expertize in violence and victimization, child and adolescent mental health, and evaluation research. She led the project, including development of the protocol, overseeing literature retrieval, participating in analysis, and leading the development of the final paper. She was supported by C. C., an expert in IPV and child maltreatment, who contributed to coding, synthesis and report writing; J. B., a social science analyst, who contributed to information retrieval, database management, citation screening and coding; and V. L. F.‐H., an expert in review methodology and statistical analysis, who provided input on all stages of the review process and conducted analyses.

### SOURCES OF SUPPORT

We received funding under the Jacobs Foundation (in partnership with the Campbell Collaboration) call for proposals, *Better Evidence for Children and Youth*.

### DECLARATIONS OF INTEREST

None of the researchers involved in the team present conflicts of interest to note.

### PLANS FOR UPDATING THE REVIEW

The lead reviewer anticipates updating the review on a 5‐year cycle, pending continued research on the topic.

## Footnotes

1 Hereafter “children” is used to refer to children and adolescents aged 0–17 years.

## APPENDIX B RISK OF BIAS ASSESSMENT

**Table 1:** Randomized controlled trials excluded after risk of bias assessment

	Selection bias			Detection bias	Performance bias	Attrition bias			Reporting bias	Other biases		
First author (publication year)	Random.	Allocation concealment	Baseline equivalence	Outcome assessors masked	Participants masked	Overall attrition	Differential attrition	Method for dropouts	Prespecified outcomes reported	Reliable and valid outcome measures	Treatment fidelity	Overall risk of bias
**Foshee (2015)**	Unclear	High	High	Unclear	High	High	Unclear	Low	Low	Low	Low	High
**Graham‐Berman (2015)**, Miller (2012), Howell (2013)	High	High	Low	Unclear	High	High	Low	Low	Low	Low	Low	High
**Graham‐Berman (2007)**	High	High	High	Unclear	High	Low	Low	High	Low	Low	High	High
**Sullivan (2002)**	Unclear	Unclear	High	Unclear	High	Low	Low	Low	Low	High	Low	High
**Kot (1995)**, Kot (1998)	High	High	High	Low	High	High	Unclear	High	Low	Mixed^a^	High	High
**Wagar (1995)**	Unclear	Unclear	Unclear	High	High	Low	Low	Unclear	Low	Low	High	High

Abbreviation: Random., randomization.

Bold denotes primary study.

^a^ Use of some outcome measures with demonstrated reliability and validity.

**Table 2:** Observational studies excluded after risk of bias assessment

	Bias in sample definition and selection						Bias in interventions		Bias in measurement of outcomes			Bias due to attrition or missing data			Bias in reporting	
First author (year)	Study design	Recruit: source	Recruit: time	Inclusion	Analyses—group differences	Bias due to confound.	Intervent. defined	Contam.	Outcome assessor masked	Outcome measures	Follow‐up	Overall attrition	Diff. attrition	Method for Dropouts	Prespecified outcomes reported	Overall risk of bias
**Pernebo (2018)**	Low	High	Low	Low	Low	High	Low	Low	Unclear	Low	Low	Low	Low	Low	Low	High
**Smith (2015)**	Low	High	Unclear	Unclear	Unclear	High	Unclear	Low	Unclear	Low	Unclear	High	Low	High	Low	High
**Waldman‐Levi (2009)**, Waldman‐Levi (2011), Waldman‐Levi (2015)	Low	High	Low	Low	High	High	Low	Low	Unclear	Low	Low	High	Low	High	Low	High
**Smith (2003)** *	High	High	High	High	High	High	Low	Low	Unclear	Low	High	High	Low	High	Low	High
**Tyndall‐Lind (2001)**	High	High	High	High	High	High	Low	Low	High	Low	Low	High	High	High	Low	High

Abbreviations: confound., counfounding; contam., contamination; diff., differential; intervent., intervention; recruit, recruitment.

Bold denotes primary study.

* Smith et al. (2003) compared intervention arms in Kot, Landreth, and Giordano (1998) and Tyndall‐Lind et al. (2001).

**Table 3:** Studies (randomized controlled trials) retained after risk of bias assessment

	Selection bias			Detection bias	Performance bias	Attrition bias			Reporting bias	Other biases		
First author (publication year)	Random.	Allocation concealment	Baseline equivalence	Outcome assessors masked	Participants masked	Overall attrition	Differential attrition	Method for dropouts	Prespecified outcomes reported	Reliable and valid outcome measures	Treatment fidelity	Overall risk of bias
**Jouriles (2001)**, McDonald (2006)	Unclear	Unclear	Low	Unclear	High	Low	Low	Unclear	Low	Low	Low	Moderate
**Lieberman (2005)**, Lieberman (2006), Ippen (2011)	Unclear	Low	Low	Low	High	Low	Low	Low	Low	Low	High	Moderate
**McFarlane (2005a)**, McFarlane (2005b)	Low	Unclear	Low	Unclear	High	Low	Low	Unclear	Low	Low	High	Moderate
**Jouriles (2009)**, McDonald (2011), Jouriles (2018)	Low	Low	Low	Unclear	High	Low	High	Low	Low	Low	High	Moderate
**Cohen (2011)**	Low	Low	Low	Low	High	High	Low	Low	Low	Low	Low	Moderate
McWhirter (2011)	Low	Low	Low	Unclear	Low	Low	Low	Unclear	Low	Mixed Low/High^a^	High	Moderate
**Overbeek (2013)**, Overbeek (2017)	Low	Low	Low	Low	Low	Low	Low	Low	Low	Low	Mixed^b^	Low
**Satyanarayana (2016)**	Low	Low	Low	Low	Low	Low	High	High	Low	Low	Mixed^c^	Moderate

Abbreviation: Random., randomization.

Bold denotes primary study.

^a^ Use of some outcome measures with demonstrated reliability and validity.

^b^ Authors reported a “limited number of deviations” from parent and child treatment manuals.

^c^ Treatment fidelity reported for only one condition.

## APPENDIX C DESCRIPTIVE TABLES

**Table 4:** Characteristics of studies included in review

First author (publication year)	Country (city or region)	Target population	Total sample *N* baseline	Total sample age range baseline (child)	Total sample % female (child) (%)	Total sample % non‐White (child) (%)	SES indicator	Key exclusion criteria
**Jouriles (2001)**, McDonald (2006)	United States (Houston‐Galveston, TX)	Mothers who reported at least one occurrence of a physically violent act from a male partner in prior 12 months, and child exhibiting clinical levels of behavior problems (*DSM‐IV* CD or ODD)	36	4–9 years	28	72	89% received public financial assistance in the past year	Mother and/or target child exhibiting symptoms of serious mental illness (e.g., psychosis, autism)
**Lieberman (2005)**, Lieberman (2006), Ippen (2011)	United States (San Francisco, CA)	Mother‐child dyads referred due to clinical concerns about child's behavior or mother's parenting after the child witnessed or overheard marital violence	75	3–5 years	52	90.7	41% with income below federal poverty line	Mother with a documented abuse of the target child, current substance abuse and homelessness, mental retardation, or psychosis. Child with mental retardation or autism spectrum disorder
**McFarlane (2005a)**, McFarlane (2005b)	United States (large urban area)	Mothers (and their child) who screened positive for past 12‐month physical or sexual IPV (any relationship to perpetrator)	258	18 months to 18 years	53.4	94.7	80.1% income <$20,000/year; 30.8% income <$5,000/year	NR
**Jouriles (2009)**, McDonald (2011), Jouriles (2018)	United States	Children exhibiting clinical levels of behavior problems (*DSM‐IV* CD or ODD) and mother reported at least one occurrence of a physically violent act from a male partner in prior 12 months	66	4–9 years	50	60.7	NR	Exclusion at prescreen: mental illness or substance use at a level that is likely to interfere with program participation; exclusion at domestic violence shelter exit screen: families moved >50 miles from the location where the project was based; mother's abusive partner lived with the family
**Cohen (2011)**	United States (Pittsburgh, PA)	Children with at least five IPV‐related PTSD symptoms and a mother who was a victim of IPV	124	7–14 years	50.8	54.4	NR	Significant developmental disorder or an IQ <80; serious psychotic symptoms in parent or child; residing in an IPV shelter
McWhirter (2011)	United States (metropolitan area in the Southwest)	Mothers with a history of past‐year IPV victimization (and scored a 15 or higher on the HITS [Hurt‐Insult‐Threaten‐Scream] IPV screening tool) and reported having a child present during 1+ IPV episode	48	6–12 years	NR	NR	NR	NR
**Overbeek (2013)**, Overbeek (2017)	The Netherlands	Parent with a history of past‐year physical and/or psychological IPV victimization	140	6–12 years	44.5	NR	66.4% with income below poverty line	Current IPV exposure; parent or child intellectual, behavioral or psychiatric problems that would impede functioning in the treatment group
**Satyanarayana (2016)**	India (Bangalore)	Married men with alcohol dependence syndrome who screened positive for past‐6‐month physical, sexual, and/or psychological IPV perpetration	177	0–16 years	NR	NR	NR	Comorbid psychosis, mood disorder or personality disorder

*Note*: Bold indicates primary study from which table information is drawn.

Abbreviations: CD, conduct disorder; ODD, oppositional defiant disorder; IPV, intimate partner violence; NR, not reported; PTSD, posttraumatic stress disorder; SES, socioeconomic status.

**Table 5:** Study characteristics: Types of IPV experienced by parent and nature of child's exposure

First author (publication year)	Exposure	Types of IPV	Victim‐perpetrator relationship
Jouriles (2001), McDonald (2006)	Inferred from mother's victimization	Physical	Mother–Male partner
Lieberman (2005), Lieberman (2006), Ippen (2011)	Mother's IPV victimization + referral source (e.g., family court, preschools) report that the child witnessed or overheard marital violence	Not defined	Mother–Husband
McFarlane (2005a, p.182), McFarlane (2005b)	Inferred from mother's victimization	Physical, sexual	Mother–Partner
Jouriles (2009), McDonald (2011), Jouriles (2018)	Inferred from mother's victimization	Physical	Mother–Male partner
Cohen (2011)	Inferred from mother's victimization	Not defined	Mother–partner
McWhirter (2011)	Mother report of child presence during at least one IPV incident	Physical, psychological	Mother–partner
Overbeek (2017, 2013)	Inferred from mother's victimization	Physical, psychological	Parent–partner
Satyanarayana (2016)	Interred from father's perpetration	Physical, sexual, psychological	Mother–Husband

**Table 6:** Characteristics of study interventions/arms included in review

First author (publication year)	Interventions and/or controls	Intervention orientation	Target of intervention	Delivery setting/modality	*N* at baseline	Frequency and duration of intervention
**Jouriles (2001)**, McDonald (2006)	Arm 1: Project Support; “intensive services” focused on child management skills and provision of emotional support	Parent training + supportive	Nonoffending parent only (mother)	Parent home	13	Weekly 90‐minute sessions for 32 weeks (mean: 23 weeks)
	Arm 2: Attention control, referral information if requested	N/A	Nonoffending parent only (mother)	Telephone or in‐person	13	Monthly contact for 32 weeks
**Lieberman (2005)**, Lieberman (2006), Ippen (2011)	Arm 1: Parent‐child psychotherapy	Play therapy	Child + parent (mother) together^a^	Outpatient clinic	36	Weekly 60‐minute sessions for 50 weeks (mean: 32 weeks)
	Arm 2: Case management + referrals for individual therapy	Supportive + unknown orientation for individual therapy	Varied; 73% of mothers and 55% of children received individual treatment	Outpatient clinic	29	Case management: monthly 30‐minute phone calls; Individual therapy: 50% of the mothers and 65% of the children receiving more than 20 individual sessions (range: 2–50 sessions for children; 6–50 sessions for mothers)
**McFarlane (2005a)**, McFarlane (2005b)	Arm 1: Screening, March of Dimes safety protocol, and nurse case management	Supportive, advocacy	Nonoffending parent only (mother)	Outpatient primary care and women, infants, and children (WIC) clinics	102 (follow‐up, baseline NR)	Four, 20‐minute sessions over 18 months + contacts as initiated by the mother; March of Dimes safety protocol provided at each session
	Arm 2: Wallet‐size information card (not part of March of Dimes protocol)	N/A	Nonoffending parent only (mother)	Outpatient primary care clinics	104 (follow‐up, baseline NR)	N/A; mother contacted only for data collection at the same four intervals as the intervention condition
**Jouriles (2009)**, McDonald (2011), Jouriles (2018)	Arm 1: Project Support; “intensive services” focused on child management skills and provision of emotional support	Parent training + supportive	Nonoffending parent only (mother)	Parent home	32	Weekly home visits for up to 8 months
	Arm 2: Attention control, referral information if requested	N/A	Nonoffending parent only (mother)	Telephone or in‐person	34	Weekly contact for 20 months (study duration)
**Cohen (2011)**	Arm 1: Trauma‐Focused Cognitive‐Behavioral Therapy (TF‐CBT)	Cognitive‐behavioral	Child + parent (mother) together	Community center	64	Weekly 45‐minute sessions for 8 weeks
	Arm 2: Child‐Centered Therapy	Play therapy	Child + parent (mother) together	Community center	60	Weekly 45‐minute sessions for 8 weeks
McWhirter (2011)	Arm 1: Goal‐oriented treatment^b^	Cognitive‐behavioral	Child + parent (mother) together	Family homeless shelter	24	Weekly 45‐minute (child) or 60‐minute (mother) sessions for 5 weeks
	Arm 2: Emotion‐focused treatment^b^	Psychoeducation	Child + parent (mother) together	Family homeless shelter	22	Weekly 45‐minute (child) or 60‐minute (mother) sessions for 5 weeks
**Overbeek (2013)**, Overbeek (2017)	Arm 1: “En nu ik…!” (It's my turn now!)^b^	Cognitive‐behavioral	Child + parent (96% mothers) separately	Community center	108	Nine, 90‐minute sessions (maximum of eight parents or children per group)
	Arm 2: “Jij hoort erbij” (You belong)^b^	Play therapy	Child + parent (94.4% mothers) separately	Community center	56	Nine, 90‐minute sessions (maximum of eight parents or children per group)
**Satyanarayana (2016)**	Arm 1: Integrated cognitive‐behavioral intervention	Cognitive‐behavioral	Perpetrating parent (father)	Inpatient psychiatric clinic	88	45‐ to 60‐minute sessions for 12 weeks
	Arm 2: Treatment as usual	Psychoeducation	Perpetrating parent (father)	Inpatient psychiatric clinic	89	45‐ to 60‐minute sessions for 12 weeks

*Note*. Bold indicates primary study from which table information is drawn.

^a^ “Parent + child together” denotes 1+ joint parent–child sessions.

^b^ Denotes intervention delivered in a group setting.

## APPENDIX D RESULTS—TABLES AND FIGURES

**Table 7:** Total behavior (CBCL) standardized effect sizes

Study: first author (year)	Intervention and comparator	Time point	Statistics for each study					
			SMD	*SE*	VAR	95% CI	*Z*	*p*
Lieberman (2005)	CPP vs. CM + individual therapy	Immediately posttreatment	0.56	0.25	0.06	−1.06 to −0.06	2.21	.03
Satyanarayana (2016)	ICBI vs. TAU	1‐month follow‐up	−0.10	0.15	0.02	−0.39 to 0.20	−0.66	.51
Satyanarayana (2016)	ICBI vs. TAU	3‐month follow‐up	−0.06	0.15	0.02	−0.35 to 0.23	−0.40	.69

Abbreviations: CBCL, Child Behavior Checklist; CPP, child‐parent psychotherapy; CI, confidence interval; CM, case management; ICBI, Integrated Cognitive Behavioral Intervention; SMD, standardized mean difference; TAU, treatment as usual; VAR, variance.

**Table 8:** Externalizing behavior standardized effect sizes

Study: first author (year)	Intervention and comparator	Time point	Outcome measure	Statistics for each study					
				SMD	*SE*	VAR	95% CI	*Z*	*p*
*Immediately posttreatment*									
Cohen (2011)	TF‐CBT vs. CCT	Immediately posttreatment	CBCL	−0.05	0.18	0.03	−0.40 to 0.30	−0.29	.78
Lieberman (2005)	CPP vs. CM + individual therapy	Immediately posttreatment	CBCL	−0.64	0.26	0.07	−1.14 to −0.14	−2.49	.01
Overbeek (2013)	Specific factors intervention vs. non‐specific factors intervention	Immediately posttreatment	CBCL	−0.08	0.18	0.03	−0.43 to 0.26	−0.46	.64
Jouriles (2001)	Intensive services vs. attention control	Immediately posttreatment	CBCL	−0.35	0.34	0.11	−1.01 to 0.31	−1.04	.30
Jouriles (2009)	Intensive services vs. attention control	Immediately posttreatment	CBCL	−0.66	0.27	0.07	−1.19 to −0.13	−2.44	.01
**Pooled Jouriles (2001) and Jouriles (2009) CBCL**	**Intensive services vs. attention control**	**Immediately posttreatment**	**CBCL**	−**0.54**	**0.21**	**0.04**	−**0.95 to** −**0.13**	−**2.55**	**.01**
Jouriles (2009)	Intensive services vs. attention control	Immediately posttreatment	ECBI	−0.17	0.27	0.07	−0.69 to 0.35	−0.64	.52
Jouriles (2009)	Intensive services vs. attention control	Immediately posttreatment	Observed oppositional behavior	−0.52	0.28	0.08	−1.07 to 0.03	−1.86	.06
Jouriles (2009)/McDonald (2011)	Intensive services vs. attention control	Immediately posttreatment	PSD	−0.24	0.25	0.06	−0.72 to 0.24	−0.97	.33
*Short‐term follow‐up*									
Jouriles (2001)	Intensive services vs. attention control	4‐month posttreatment	CBCL	−0.12	0.33	0.11	−0.77 to 0.54	−0.35	.72
Jouriles (2009)/Jouriles (2018)	Intensive services vs. attention control	4‐month posttreatment	CBCL	−0.18	0.25	0.06	−0.66 to 0.30	−0.73	.47
**Pooled Jouriles (2001) and Jouriles (2009) CBCL**	**Intensive services vs. attention control**	**4‐month posttreatment**	**CBCL**	−**0.16**	**0.20**	**0.04**	−**0.55 to 0.23**	−**0.80**	.43
Overbeek (2013)	Specific factors intervention vs. nonspecific factors intervention	6‐month posttreatment	CBCL	−0.30	0.18	0.03	−0.65 to 0.05	−1.68	.09
Lieberman (2005)	CPP vs. CM + individual therapy	6‐month posttreatment	CBCL	−0.70	0.29	0.09	−1.27 to −0.12	−2.38	.02
Jouriles (2001)	Intensive services vs. attention control	8‐month posttreatment	CBCL	−0.82	0.35	0.12	−1.50 to −0.14	−2.36	.02
Jouriles (2009)/Jouriles (2018)	Intensive services vs. attention control	8‐month posttreatment	CBCL	−0.70	0.25	0.06	−1.20 to −0.21	−2.77	.01
**Pooled Jouriles (2001) and Jouriles (2009) CBCL**	**Intensive services vs. attention control**	**8‐month posttreatment**	**CBCL**	−**0.74**	**0.20**	**0.04**	−**1.15 to** −**0.34**	−**3.63**	**.09**
*Long‐term follow‐up*									
Jouriles (2009)/Jouriles (2018)	Intensive services vs. attention control	12‐month posttreatment	CBCL	−0.33	0.25	0.06	−0.82	0.16	.18
Jouriles (2009)	Intensive services vs. attention control	12‐month posttreatment	ECBI	−0.66	0.31	0.09	−1.26	−0.06	.03
Jouriles (2009)/Jouriles (2018)	Intensive services vs. attention control	16‐month posttreatment	CBCL	−0.58	0.25	0.06	−1.08	−0.09	.02
Jouriles (2009)	Intensive services vs. attention control	16‐month posttreatment	Observed oppositional behavior	−0.57	0.30	0.09	−1.15	0.01	.05
Jouriles (2009)/Jouriles (2018)	Intensive services vs. attention control	20‐month posttreatment	CBCL	−0.65	0.25	0.06	−1.15	−0.16	.01
Jouriles (2001)/McDonald (2006)	Intensive services vs. attention control	24‐month posttreatment	CBCL	−0.93	0.44	0.20	−1.80	−0.06	.04

*Note*: Bold indicates meta‐analysis of two studies.

Abbreviations: CCT, child centered therapy; CPP, child‐parent psychotherapy; CI, confidence interval; CM, case management; SMD, standardized mean difference; TAU, treatment as usual; TF‐CBT, trauma focused cognitive behavioral therapy; VAR, variance.

**Table 9:** Internalizing behavior standardized effect sizes

Study: first author (year)	Intervention and comparator	Time point	Outcome measures	Statistics for each study					
				SMD	SE	VAR	95% CI	*Z*	*p*
Jouriles (2001)	Intensive Services vs. Attention Control	Immediately posttreatment	CBCL	−0.68	0.34	0.12	−1.36 to −0.01	−2.00	.05
McWhirter (2011)	Goal‐oriented Intervention vs. Emotion‐focused Intervention	Immediately posttreatment	Visuo‐graphically emotional barometer	−0.01	0.30	0.09	−0.59 to 0.57	−0.03	.98
Overbeek (2013)	Specific Factors Intervention vs. Non‐specific Factors Intervention	Immediately posttreatment	CBCL	−0.22	0.18	0.03	−0.56 to 0.13	−1.23	.22
Jouriles (2001)	Intensive Services vs. Attention Control	4‐month follow‐up	CBCL	−0.14	0.33	0.11	−0.79 to 0.52	−0.41	.68
Overbeek (2013)	Specific Factors Intervention vs. Non‐specific Factors Intervention	6‐month follow‐up	CBCL	−0.27	0.18	0.03	−0.62 to 0.08	−1.53	.13
Jouriles (2001)	Intensive Services vs. Attention Control	8‐month follow‐up	CBCL	−0.80	0.35	0.12	−1.48 to −0.12	−2.31	.02
Jouriles (2001)/McDonald (2006)	Intensive Services vs. Attention Control	24‐month follow‐up	CBCL	−1.50	0.84	0.70	−3.14 to 0.15	−1.78	.07

Abbreviations: CI, confidence interval; SMD, standardized mean difference; VAR, variance.

**Table 10:** Posttraumatic stress standardized effect sizes

Study: first author (year)	Intervention and comparator	Time point	Outcome measures	Statistics for each study					
				SMD	SE	VAR	95% CI	*Z*	*p*
Lieberman (2005)	CPP vs. CM + individual therapy	Immediately posttreatment	PTSD (DC 0–3)	0.87	0.26	0.07	0.36 to 1.38	3.32	.00
Overbeek (2013)	Specific factors intervention vs. nonspecific factors intervention	Immediately posttreatment	TSCC	0.32	0.22	0.05	−0.12 to 0.76	1.43	.15
Overbeek (2013)/Overbeek (2017)	Specific factors intervention vs. nonspecific factors intervention	6‐month follow‐up	TSCYC	−0.69	0.19	0.03	−1.06 to −0.33	−3.70	.00
Overbeek (2013)	Specific factors intervention vs. nonspecific factors intervention	6‐month follow‐up	TSCC	−0.13	0.22	0.05	−0.56 to 0.31	−0.56	.58

Abbreviations: CPP, child‐parent psychotherapy; CI, confidence interval; CM, case management; DC 0–3, Diagnostic Classification of Mental Health and Developmental Disorders of Infancy and Early Childhood; PTSD, posttraumatic stress disorder; SMD, standardized mean difference; TAU, treatment as usual; TSCC, Trauma Symptom Checklist for Children; TSCYC, Trauma Symptom Checklist for Young Children; VAR, variance

**Table 11:** Anxiety, depression, cognitive ability, and happiness/social relationships standardized effect sizes

Study: First author (year)	Intervention and comparator	Time point	Outcome measures	Statistics for each study					
				SMD	SE	VAR	95% CI	*Z*	*p*
Cohen (2011)	TF‐CBT vs. CCT	Immediately posttreatment	Anxiety (SCARED)	−0.37	0.18	0.03	−0.72 to −0.01	−2.04	.04
Cohen (2011)	TF‐CBT vs. CCT	Immediately posttreatment	Cognitive ability (KBIT)	0.37	0.18	0.03	0.02 to 0.73	2.05	.04
Cohen (2011)	TF‐CBT vs. CCT	Immediately posttreatment	Depression (CDI)	−0.21	0.18	0.03	−0.57 to 0.14	−1.19	.23
Overbeek (2013)	Specific factors intervention vs. nonspecific factors intervention	Immediately posttreatment	Depression (CDI)	−0.11	0.20	0.04	−0.49 to 0.28	−0.55	.58
Overbeek (2013)	Specific factors intervention vs. nonspecific factors intervention	6‐month follow‐up	Depression (CDI)	−0.21	0.18	0.03	−0.57 to 0.14	−1.19	.23
Jouriles (2001)/McDonald (2006)	Intensive services vs. attention control	24‐month follow up	Happiness/social relationships (drawn from HRI)	0.53	0.37	0.14	−0.21 to 1.26	1.40	.16

Abbreviations: CDI, Children's Depression Inventory; HRI, Health Resources Inventory; KBIT, Kaufman Brief Intelligence Test; SCARED, Screen for Child Anxiety Related Emotional Disorder; CI, confidence interval; SE, standard error; SMD, standardized mean difference; VAR, variance

**Table 12:** Differences in standardized effect sizes between a nurse‐lead case management intervention and inactive control by age group

Subgroup	Time point	Statistics for each study					
		SMD	SE	VAR	95% CI	*Z*	*p*
*McFarlane (2005a)—total problems (CBCL)*							
Older age	6‐month follow‐up	−0.03	0.18	0.03	−0.37 to 0.32	−0.16	.87
Younger age	6‐month follow‐up	−0.24	0.20	0.04	−0.63 to 0.15	−1.22	.22
Older age	12‐month follow up	−0.04	0.18	0.03	−0.38 to 0.30	−0.22	.82
Younger age	12‐month follow up	−0.13	0.20	0.04	−0.52 to 0.26	−0.66	.51
Older age	18‐month follow‐up	−0.28	0.18	0.03	−0.62 to 0.07	−1.57	.12
Younger age	18‐month follow‐up	−0.02	0.20	0.04	−0.41 to 0.37	−0.10	.92
Older age	24‐month follow‐up	−0.30	0.18	0.03	−0.64 to 0.05	−1.69	.09
Younger age	24‐month follow‐up	−0.01	0.20	0.04	−0.39 to 0.38	−0.04	.97
*McFarlane (2005)—externalizing problems (CBCL)*							
Older age	6‐month follow‐up	−0.08	0.18	0.03	−0.42 to 0.26	−0.46	.65
Younger age	6‐month follow‐up	−0.19	0.20	0.04	−0.58 to 0.20	−0.96	.34
Older age	12‐month follow up	−0.10	0.18	0.03	−0.44 to 0.24	−0.57	.57
Younger age	12‐month follow up	−0.11	0.20	0.04	−0.50 to 0.27	−0.57	.57
Older age	18‐month follow‐up	−0.07	0.18	0.03	−0.41 to 0.28	−0.38	.70
Younger age	18‐month follow‐up	−0.05	0.20	0.04	−0.43 to 0.34	−0.24	.81
Older age	24‐month follow‐up	−0.19	0.20	0.04	−0.58 to 0.20	−0.96	.34
Younger age	24‐month follow‐up	−0.05	0.20	0.04	−0.43 to 0.34	−0.23	.81
*McFarlane (2005)—internalizing problems (CBCL)*							
Older age	6‐month follow‐up	−0.12	0.18	0.03	−0.47 to 0.22	−0.69	.49
Younger age	6‐month follow‐up	−0.21	0.20	0.04	−0.59 to 0.18	−1.05	.29
Older age	12‐month follow up	−0.12	0.18	0.03	−0.46 to 0.23	−0.66	.51
Younger age	12‐month follow up	−0.20	0.20	0.04	−0.58 to 0.19	−1.00	.32
Older age	18‐month follow‐up	−0.13	0.18	0.03	−0.47 to 0.22	−0.73	.46
Younger age	18‐month follow‐up	−0.03	0.20	0.04	−0.41 to 0.36	−0.14	.89
Older age	24‐month follow‐up	−0.16	0.18	0.03	−0.50 to 0.19	−0.91	.36
Younger age	24‐month follow‐up	−0.01	0.20	0.04	−0.39 to 0.38	−0.04	.97

Abbreviations: CI, confidence interval; SMD, standardized mean difference; VAR, variance.

**Table 13:** Differences in standardized effect sizes between child‐parent psychotherapy (CPP) and case management (CM) plus individual therapy by number of traumatic and stressful life events

Subgroup	Time point	Outcome construct (measure)	Statistics for each study					
			SMD	SE	VAR	95% CI	*Z*	*p*
*Lieberman (2005)/Ippen (2011)*								
High risk	Immediately posttreatment	Total behavior (CBCL)	−1.14	0.37	0.14	−1.86 to −0.42	−3.10	.00
Low risk	Immediately posttreatment	Total behavior (CBCL)	−0.01	0.32	0.10	−0.64 to 0.61	−0.04	.97
High risk	6‐month follow‐up	Total behavior (CBCL)	−1.66	0.39	0.16	−2.43 to −0.88	−4.20	.00
Low risk	6‐month follow‐up	Total behavior (CBCL)	−0.15	0.32	0.10	−0.77 to 0.48	−0.46	.64
High risk	Immediately posttreatment	PTSD (DC 0–3)	−1.65	0.39	0.16	−2.42 to −0.87	−0.87	−4.18
Low risk	Immediately posttreatment	PTSD (DC 0–3)	−0.33	0.32	0.10	−0.96 to −0.30	0.30	−1.03
High risk	Immediately posttreatment	Depression (DC 0–3)	−0.99	0.36	0.13	−1.70 to −0.28	−2.73	.01
Low risk	Immediately posttreatment	Depression (DC 0–3)	−0.02	0.32	0.10	−0.64 to 0.61	−0.05	.96

*Note*. High risk, youth with four or more lifetime exposures to traumatic and stressful life events; Low Risk, Youth with less than four lifetime exposures to traumatic and stressful life events.

Abbreviations: CPP, child‐parent psychotherapy; CI, confidence interval; CM, case management; DC 0–3, Diagnostic Classification of Mental Health and Developmental Disorders of Infancy and Early Childhood; SMD, standardized mean difference; TSE, traumatic and stressful events; VAR, variance.

**Table 14:** Standardized externalizing CBCL scores by modality immediately posttreatment

Modality	Study first author (year)	Comparison	Statistics for each study					
			SDM	SE	VAR	95% CI	*Z*	*p*
Family together	Cohen (2011)	TF‐CBT vs. CCT	−0.05	0.18	0.03	−0.40 to 0.30	−0.29	.78
Family together	Lieberman (2005)	CPP vs. CM + Individual therapy	−0.64	0.26	0.07	−1.14 to −0.14	−2.49	.01
Family together			−**0.25**	**0.15**	**0.02**	−**0.53 to 0.04**	−**1.67**	**.10**
Non‐offending parent (mother)	Jouriles (2009)	Intensive Services vs. Attention Control	−0.66	0.27	0.07	−1.19 to −0.13	−2.44	.01
Non‐offending parent (mother)	Jouriles (2001)	Intensive Services vs. Attention Control	−0.35	0.34	0.11	−1.01 to 0.31	−1.04	.30
Non‐offending parent (mother)			−**0.38**	**0.12**	**0.02**	−**0.62 to** −**0.14**	−**3.09**	**.00**
Parent and child separate	Overbeek (2013)	Specific Factors Intervention vs. Non‐specific Factors Intervention	−0.08	0.18	0.03	−0.43 to 0.26	−0.46	.64
Parent and child separate			−**0.08**	**0.18**	**0.03**	−**0.43 to 0.26**	−**0.46**	**.64**
Overall			−**0.26**	**0.10**	**0.01**	−**0.45 to** −**0.06**	−**2.60**	**.01**

Abbreviations: CCT, Child Centered Therapy; CPP, Child‐Parent Psychotherapy; CI, confidence interval; CM, Case Management; SMD, standardized mean difference; TF‐CBT, trauma focused cognitive behavioral therapy; VAR, variance.

**Table 15:** Standardized externalizing CBCL scores by setting immediately posttreatment

Setting	Study first author (year)	Comparison	Statistics for each study					
			SDM	SE	VAR	95% CI	*Z*	*p*
Home	Jouriles (2009)	Intensive services vs. attention control	−0.66	0.27	0.07	−1.19 to −0.13	−2.44	.01
Home	Jouriles (2001)	Intensive services vs. attention control	−0.35	0.34	0.11	−1.01 to 0.31	−1.04	.30
Home			−**0.54**	**0.21**	**0.04**	−**0.95 to** −**0.13**	−**2.55**	**.01**
OP	Cohen (2011)	TF‐CBT vs. CCT	−0.05	0.18	0.03	−0.40 to 0.30	−0.29	.78
OP	Lieberman (2005)	CPP vs. CM + individual therapy	−0.64	0.26	0.07	−1.14 to −0.14	−2.49	.01
OP	Overbeek (2013)	Specific factors intervention vs. nonspecific factors intervention	−0.08	0.18	0.03	−0.43 to 0.26	−0.46	.64
OP			−**0.18**	**0.11**	**0.01**	−**0.40 to 0.04**	−**1.58**	**.11**
Overall			−**0.26**	**0.10**	**0.01**	−**0.45 to** −**0.06**	−**2.60**	**.01**

Abbreviations: CCT, child centered therapy; CPP, child‐parent psychotherapy; CI, confidence interval; CM, case management; SMD, standardized mean difference; TF‐CBT, trauma focused cognitive behavioral therapy; VAR, variance.
